# Supplementary material for: Patch deconvolution for Fourier light-field microscopy
Source: Biophys J. 2026 Jan 21;125(5):1305–14. doi: 10.1016/j.bpj.2026.01.034 (PMC13351718; doi:10.1016/j.bpj.2026.01.034)
Supplement: Document S1. Figures S1–S14, Tables S1 and S2, and Sections S1–S6 [file mmc1.pdf]

Biophysical Journal, Volume *125*

Supplemental information

Patch deconvolution for Fourier light-field microscopy

Bin Fu, Caroline L. Jones, Daniel Heraghty, Shengbo Yang, Caitlin O'Brien-Ball, Victoria Junghans, Haowei Yang, David Klenerman, Tuomas P.J. Knowles, Lucien E. Weiss, Ricardo A. Fernandes, and Steven F. Lee

Manuscript submitted to **Biophysical** *Journal*
Article

Supplementarty Information: Patch deconvolution for Fourier light-field microscopy

Bin Fu[1], Caroline L. Jones[1], Daniel Heraghty[1], Shengbo Yang[1], Caitlin O'Brien-Ball[2], Victoria Junghans[2], Haowei Yang[1], David Klenerman[1], Tuomas P.J. Knowles[1], Lucien E. Weiss[3], Ricardo A. Fernandes[2], and Steven F. Lee[1,*]

[1]Yusuf Hamied Department of Chemistry, University of Cambridge, Lensfield Road, Cambridge, CB2 1EW, UK
[2]Chinese Academy of Medical Sciences (CAMS) Oxford Institute (COI), University of Oxford, Oxford, OX3 7BN, UK
[3]Department of Engineering Physics, Polytechnique Montréal, Montréal, Québec, H3T 1J4, Canada
[*]Correspondence: sl591@cam.ac.uk

S1 OPTICAL SYSTEM DESIGN

S1.1 System schematic

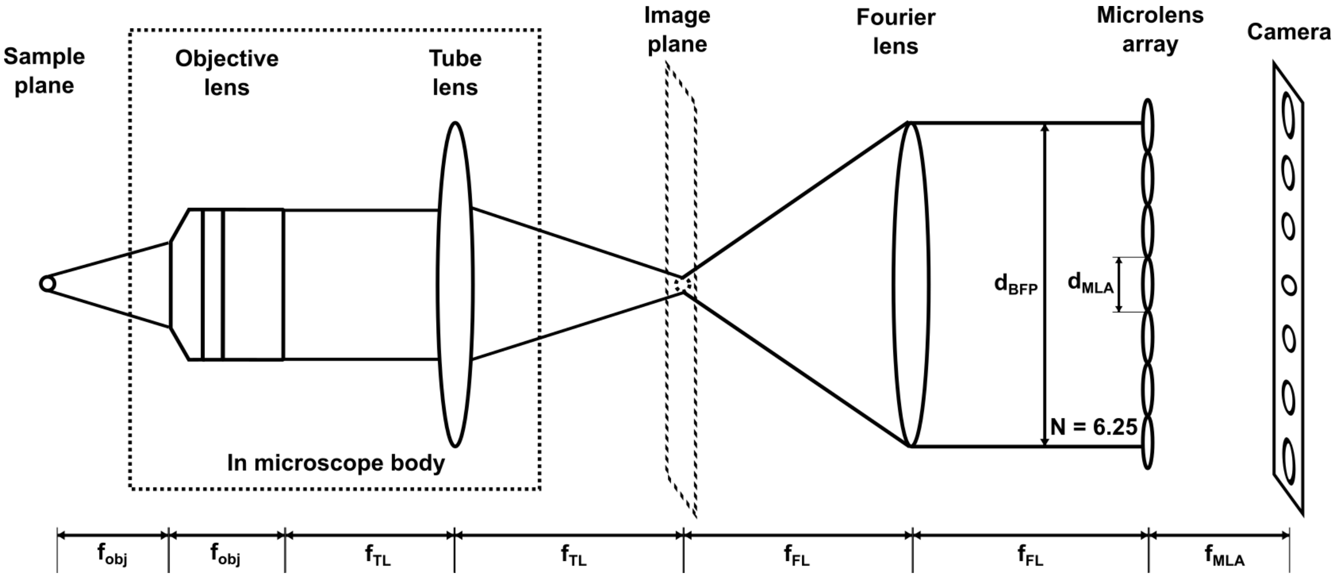

Figure S1: **Schematic layout of FLFM.** The simplified system of a FLFM microscope with objective lens, tube lens, Fourier lens, MLA and the camera. Multiple perspectives of the sample are formed on the camera due to the MLA. N = 6.25 is the setup for 37 MLA included in the back focal plane (BFP)

The lateral resolution, defined in Eqn. S1, and the axial resolution, defined in Eqn. S2 (1), are two key parameters in the FLFM system design, where N is the ratio between the diameter of back focal plane (BFP) (d_{BFP}) and the diameter of a microlens (d_{MLA}) and NA represents the objective lens's numerical aperture. Since the diameter of BFP is divided by N microlenses, the effective numerical aperture ($\mathrm{NA}_{\mathrm{MLA}}$) for each perspective image in FLFM can be described as NA/N. Therefore, the lateral resolution can be represented in S1.

$$R_{\mathrm{xy}} = \frac{\lambda}{2\mathrm{NA}_{\mathrm{MLA}}} = \frac{\lambda N}{2\mathrm{NA}} \tag{S1}$$

The equation for axial resolution, derived from ray optics, has been adapted for a hexagonal microlens array (MLA) pattern. In the case of a 37 MLA configuration (Fig. S1), the transformation from axial displacement in the sample plane to lateral displacement due to MLA on the camera is represented by $\tan\theta$ (1). Here, θ is the angle formed between f_{FL} and half the

radius of the BFP after the Fourier lens. This angle represents the average axial shift across all perspectives on camera. Hence, $\tan\theta = \frac{N}{4} \times \frac{d_{\mathrm{MLA}}}{f_{\mathrm{FL}}}$. The axial resolution can be determined by back-projecting the lateral resolution to its corresponding axial distance in the sample plane, *i.e.*the minimum axial separation at which two emitters can be resolved. This minimum distance can be calculated using Eqn. S2 using $d_{\mathrm{MLA}} = 2f_{\mathrm{MLA}}\mathrm{NA}_{\mathrm{MLA}}$

$$R_z = \frac{\lambda}{2\mathrm{NA}_{\mathrm{MLA}}} \times \frac{f_{\mathrm{FL}}}{f_{\mathrm{MLA}}} \times \frac{1}{\tan\theta} \times \left(\frac{f_{obj}}{f_{TL}}\right)^2 = \frac{\lambda N}{\mathrm{NA}^2} \quad \text{(S2)}$$

The depth-of-field (DoF) defined in Eqn. S3 is also an important factor in the system design. It is described by the sum of the wave and geometrical optical depths of fields where δ is the pixel size of the camera and M_{T} is the total magnification of the system (2).

$$DoF = 2\lambda\frac{N^2}{\mathrm{NA}^2} + \frac{\delta}{M_{\mathrm{T}}}\frac{N}{\mathrm{NA}} \quad \text{(S3)}$$

The field-of-view (FoV) is another crucial consideration in system design to ensure complete cell capture, which is determined by M_{T} and the pitch of MLA.

$$\mathrm{FoV} = \frac{d_{\mathrm{MLA}}}{M_{\mathrm{T}}} \quad \text{(S4)}$$

To image an entire cell in flow, several design requirements must be met. First, lateral needs to be within 1-2 µm while axial resolution needs to be within 2-3 µm to achieve similar lateral and axial resolution sufficient for capturing cell morphology. This requirement sets an upper limit on N in BFP. Second, the DoF must exceed 15 µm to image the whole cell while allowing for some movement within the microfluidic channel, establishing a lower limit on N in BFP. Based on these constraints, we validated the patch deconvolution using 19 MLA and 37 MLA setup.

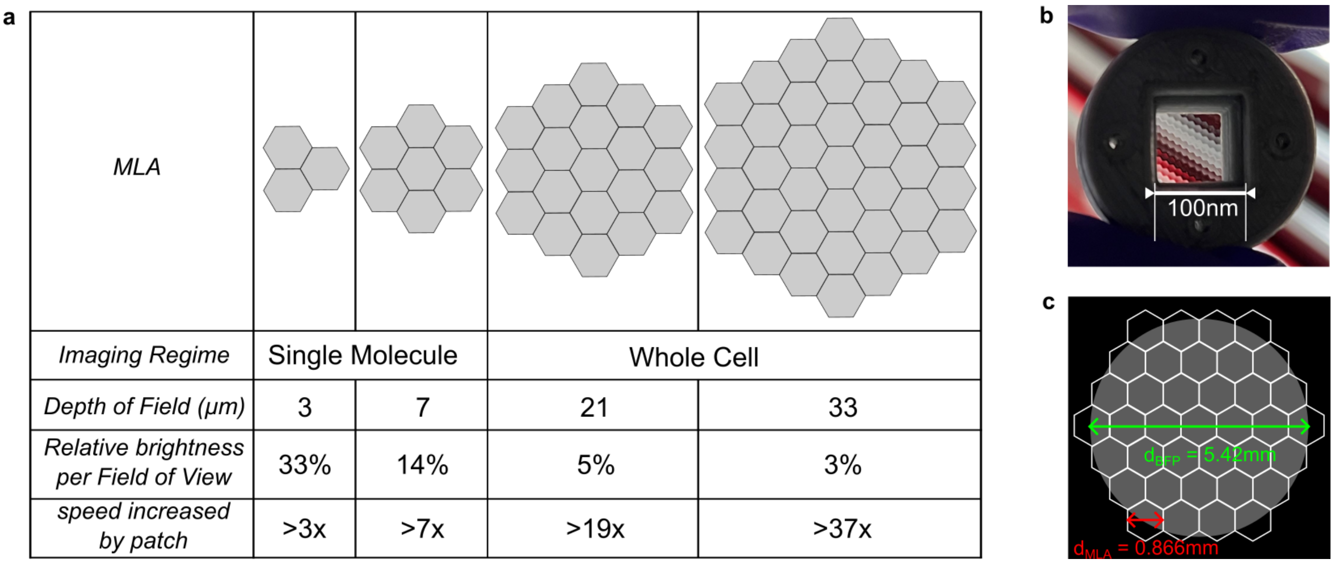

Figure S2: **Parameter comparison. a** Diagram illustrating different configurations of MLA used for various imaging regimes, including Single Molecule and Whole Cell imaging. By varying in size and number, the depth of field, relative brightness per field of view, and speed increase achieved by patching are different for different configurations. **b** Photograph of MLA in SM1 compatible retaining ring. **c** Depiction of BFP size and the number of MLA contained within. Fourier lens of 125 mm produces BFP diameter of 5.42 mm covering 6.25 lenses for 37 MLA setup.

S1.2 System design parameters

The specific design parameters for 19 MLA and 37 MLA setup is listed in the Table S1. The only change between two setups is the choice of Fourier lens: 100 mm for a 19 MLA setup and 125 mm for a 37 MLA setup, which leads to a different BFP size (4.33 mm for 19 MLA setup and 5.42 mm for 37 MLA setup).

Variable	Symbol	Value (mm)
Numerical aperture	NA	1.3
Objective magnification	M_{obj}	60
Tube lens focal length (mm)	f_{TL}	180
Objective focal length (mm)	$f_{\text{obj}} = \frac{f_{\text{TL}}}{M_{\text{obj}}}$	3.0
Fourier lens focal length (mm)	f_{FL}	100/125
MLA focal length (mm)	f_{MLA}	36.7
MLA height (mm)	d_{MLA}	0.866
Pixel size (µm)	δ	6.5
Emission wavelength (nm)	λ	680
Total magnification	$M_{\text{T}} = \frac{f_{\text{MLA}} f_{\text{TL}}}{f_{\text{obj}} f_{\text{FL}}}$	22/17.6
Diameter BFP (mm)	$d_{\text{BFP}} = \frac{2\text{NA} f_{\text{obj}} f_{\text{FL}}}{f_{\text{TL}}}$	4.33/5.42
Number of MLA in BFP	$N = \frac{d_{\text{BFP}}}{d_{\text{MLA}}}$	5/6.25

Table S1: **Summary of design parameters in the FLFM optical system design.** The first column shows the parameter from 19 MLA setup while the second column shows the parameters from 37 MLA setup.

With such design parameters and using the equations discussed in the previous section, the performance parameter can be concluded in the Table S2 where the first column corresponds to 19 MLA setup and the second corresponds to the 37 MLA setup

Variable	Symbol	Value (mm)
Lateral resolution (µm)	R_{xy}	1.3/1.6
Axial resolution (µm)	R_{z}	2.0/2.6
Depth of field (µm)	DoF	21.2/33.2
Field Of View (µm)	FoV	39.4/49.2

Table S2: **Summary of performance of the system based on the design parameters.** The first column shows the parameter from 19 MLA setup while the second column shows the parameters from 37 MLA setup.

S2 IMAGE FORMATION AND RECONSTRUCTION MODEL

S2.1 Forward imaging model

The forward projection model is essential for simulating FLFM images. In this paper, the forward projection model is derived from the approach presented in (3).

Initially, the phase $\Phi(u, v)$ at the BFP, resulting from displacements of a point source from the focal point, is given by Eqn. S5. (x,y,z) denotes the coordinate at the object plane, and (u, v) denotes the coordinate at the BFP. n_s is the refractive index of the sample. k is the wavevector defined as $k = \frac{2\pi}{\lambda_0}$, and f is the focal length of the objective lens. Here, (x, y) in the BFP plane is mapped directly to (u, v) in the camera plane, since the ray height is preserved during the mapping from the object plane to the BFP plane (4).

$$\Phi(u, v; x, y, z) = \frac{n_s k}{f} \left[(xu + yv) + z\sqrt{f^2 - u^2 - v^2} \right] \tag{S5}$$

In the normalized coordinate system at the BFP, the normalized radius, ρ, is defined such that $\rho^2 = u^2 + v^2 = 1$. According to the small angle approximation, the normalized radius of the BFP is approximately equal to $f \cdot \text{NA}$. When accounting for the mismatch between the sample's refractive index and the objective lens, the focal length can be derived as $f = n_s/\text{NA}$. Therefore, Eqn. S5 becomes:

$$\Phi(u, v; x, y, z) = k\text{NA}\,(xu + yv) + n_s z k \sqrt{1 - \left(\frac{\text{NA}\rho}{n_s}\right)^2} \tag{S6}$$

By introducing a microlens array at the BFP, which acts as a phase modulator, the phase $\Phi(u, v)$ in Eqn. S6 is modified to Eqn. S7. In this equation, $\Phi_{mla}(u, v)$ represents the extra phase from the microlens array.

$$\Phi'(u, v; x, y, z) = \Phi(u, v; x, y, z) + \Phi_{mla}(u, v) \tag{S7}$$

The electric field $E(x, y)$ at the camera can be modeled using Fresnel propagation over the distance f_{mla} from the BFP. The square of the magnitude of the electric field $E(x, y)$, assuming circularly polarised, is the point spread function (PSF) $h(x, y)$ of the system, described in Eqn. S8. Here, (u, v) in the BFP plane is mapped directly to (x, y) in the camera plane (4)

$$h(u, v; x, y, z) = \left\{\mathcal{F}^{-1}\left\{\mathcal{F}\{\exp(\Phi'(u, v; x, y, z))\} \cdot \exp\left(\frac{-j}{4\pi}\lambda f_{mla}(u^2 + v^2)\right)\right\}\right\}^2 \tag{S8}$$

Since FLFM is spatially invariant (5), the PSF $h(u, v; x, y, z)$ can be simplified to $h(u, v; z)$ by ignoring kNA $(xu + yv)$ term in Eqn. S6, where z denotes the location of the point source along the z-axis. Based on this simplified $h(u, v)$, and the corresponding coordinate between sample plane (x, y) and the image plane (u, v), the forward model of FLFM can be represented by Eqn. S9, where $o(x, y; z)$ represents the object distribution function $o(x, y)$ at a specific z-plane and $I(x, y)$ represents the simulated FLFM image.

$$I(x, y) = \int_z h(x, y; z) * o(x, y; z) dz \tag{S9}$$

S2.2 Richardson-Lucy deconvolution for FLFM

To solve this inverse problem (*i.e.*recovering the object from the observed image), we begin with the simplest case where both the observed image $I(x, y)$ and the object $o(x, y)$ are 2D images. Since the noise statistics of an image are dominated by a Poisson process (6), the distribution P of an observed image I at pixel s can be expressed in Eqn. S10. Here, the forward imaging model is represented as $(o * h)(s)$, where s represents the coordinates (x, y).

$$P(I(s)|(o * h)(s)) = \frac{[(o * h)(s)]^{I(s)} e^{-(o*h)(s)}}{I(s)!} \tag{S10}$$

Assuming that the noise is spatially uncorrelated, the likelihood distribution for all pixels in the observed image I, given the object o, can be expressed in Eqn. S11 where S represents the total set of pixel coordinates in the image.

$$P(I|o) = \prod_{s \in S} \left(\frac{[(o * h)(s)]^{I(s)} e^{-(o*h)(s)}}{I(s)!}\right) \tag{S11}$$

To maximize the likelihood distribution in Eqn. S11 with respect to o, we can transform Eqn. S11 to $-\log(P(I|o))$. This transformation converts the maximization problem into a minimization problem, where the objective is to minimize $J_1(o)$, as defined in Eqn. S12.

$$J_1(o) = \sum_s (-I(s) \cdot \log[(o * h)(s)] + (o * h)(s)) \tag{S12}$$

To find the optimal solution for $J_1(o)$, it is equivalent to solve $\frac{\partial J_1(o)}{\partial o} = 0$. By using $g(o)$ representing $(o * h)(s)$, $\frac{\partial J_1(o)}{\partial o} = \frac{\partial J_1(g)}{\partial g} \cdot \frac{\partial g(o)}{\partial o}$. The $\frac{\partial J_1(o)}{\partial o} = 0$ therefore can be expressed in Eqn. S13

$$\frac{I(s)}{g(s)} * h(-s) = \sum_{s \in S} h(s) \tag{S13}$$

Since the PSF h also represents as the probability distribution of a single photon, the right hand side of Eqn. S13 is 1. Therefore, Eqn. S13 can be converted to Eqn. S14

$$\frac{I(s)}{(o * h)(s)} * h(-s) = 1 \tag{S14}$$

To solve Eqn. S14, the iterative maximum-likelihood by expectation maximization (MLEM) algorithm from Richardson (7) and Lucy (8) is given by Eqn. S15 where k represents the iteration index.

$$o_{k+1}(s) = \left\{ \left[\frac{I(s)}{(o_k * h)(s)} \right] * h(-s) \right\} \cdot o_k(s) \tag{S15}$$

The Richardson-Lucy deconvolution (Eqn. S15) in FLFM can be adapted in Eqn. S16

$$o_{k+1}(x,y,z) = \left\{ \left[\frac{I(x,y;z)}{\int_z h(x,y;z) * o(x,y;z)dz} \right] * h(-x,-y;z) \right\} \cdot o_k(x,y,z) \tag{S16}$$

S2.3 Patch deconvolution for FLFM

The major difference between RL deconvolution and patch deconvolution lies in the size of the PSF h, the FLFM image I, and the object o used during the iterative process. In patch deconvolution, the PSF h and the FLFM image I are cropped, resulting in the object also being cropped to match the size of the FLFM or the PSF patch. Consequently, the coordinates in the image, originally denoted by (x, y), are changed to (x_p, y_p), which represent local coordinates in the patch. Thus, Eqn. S16 is modified to Eqn. S17 for patch deconvolution where I_p and h_p represents the pth patch of FLFM image and PSF.

$$o_{k+1}(x_p,y_p,z) = \left\{ \left[\frac{I_p(x_p,y_p;z)}{\int_z o_k(x_p,y_p;z) * h_p(x_p,y_p;z)dz} \right] * h_p(-x_p,-y_p;z) \right\} \cdot o_k(x_p,y_p,z) \tag{S17}$$

In RL deconvolution, a single PSF is used only once, whereas in patch deconvolution, it can be utilized p times, where p is the number of perspectives (*i.e.*patches) in the FLFM image. This repeated use of the same PSF information leads to more frequent updates, resulting in a much faster convergence speed for patch deconvolution compared to RL deconvolution. The patch deconvolution algorithm is fully compatible with the existing RL code. The main adjustment involves changing the input from a single PSF and image to different patches of the image and their corresponding PSFs. The algorithm can be implemented using the existing RL algorithm as follows:

(1). **Patch Extraction**: Crop each view in the image and their corresponding PSFs. The result should be a 4D matrix (m, n, z, p), where (m,n) is the size of each patch, z represents the sampling range along the z-axis, and p is the number of perspectives.

(2). **Initialization**: Initialize the object volume (m, n, z) with all elements set to one.

(3). **Iterative Update**: Call the RL deconvolution function, but use a randomly chosen patch image and the corresponding patch PSF as inputs. Also, set the number of iterations to 1. Then, use another randomly selected patch of image and PSF, along with the updated volume from the previous step, for the next iteration.

(4). **Repeat Iterations**: Repeat step (3) until all patches have been utilized. After completing a pass through all patches, continue iterating by revisiting patches as needed. The iterations can be stopped at any point based on a convergence criteria or after a predetermined number of iterations.

S2.4 Convergence of patch deconvolution

The derivation is based on the work of Broxton (9) and Hudson (10).

As described by Broxton (9), the PSF h in the image formation model without considering noise $I = o * h$ can be discretised and the model can be reformulated as a linear system $y = Hx$, where H is the projection matrix whose elements H_{tj} represent the proportion of light from voxel t in the object volume reaching pixel j in the FLFM image. The FLFM image has the number of pixels $N_x \times N_y$, while the object has the number of voxels $N_x \times N_y \times N_z$. The vectors y and x are the flattened forms of the FLFM image and object volume, respectively.

RL deconvolution is an iterative maximum-likelihood estimation algorithm for finding a feasible solution for x. For the model $y = Hx$, the multiplicative update rule at iteration k can be written as:

$$x_j^{k+1} = x_j^k \sum_{t \in N_p} \frac{y_t}{\mu_t^k} H_{tj}, \quad \text{where} \quad \mu_t^k = \sum_{j \in N_v} H_{tj} x_j^k \tag{S18}$$

In patch deconvolution, only a subset $S_k \subset N_p$ is used at each iteration k, leading to the update:

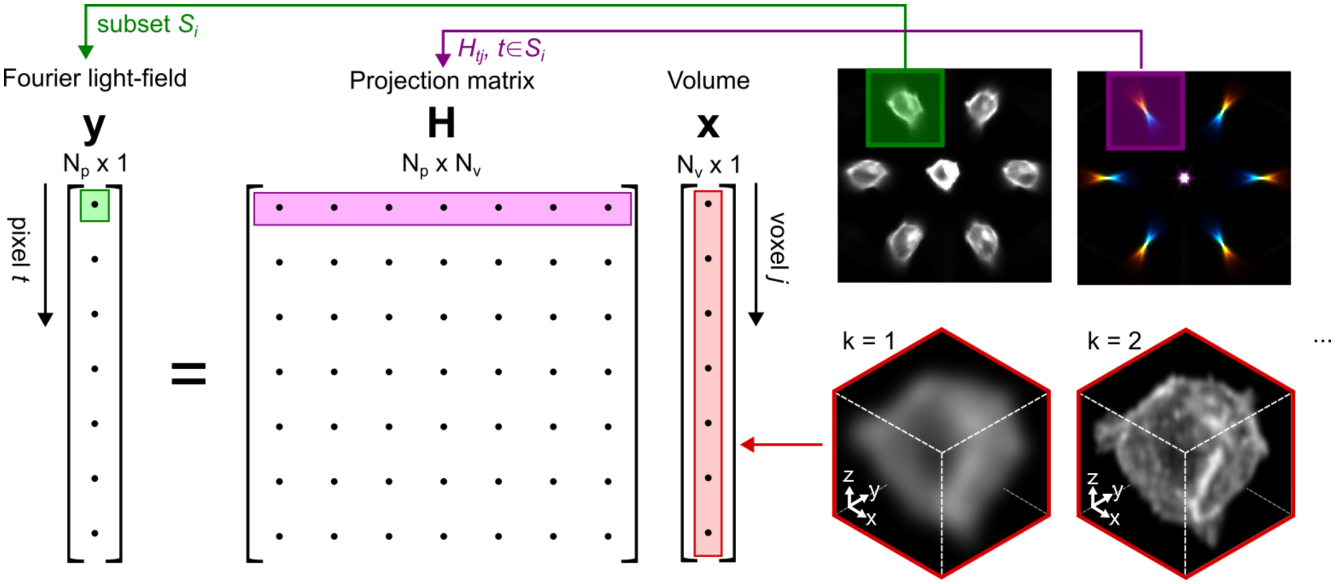

Figure S3: **The discrete patch deconvolution model without noise.** The FLFM image is flattened into a column vector of length $N_p \times 1$, where $N_p = N_x \times N_y$. Each subset S corresponds to a specific group of rows in this vector (*i.e.* $t \in S_i$). Similarly, the object volume is flattened into a vector of length $N_v \times 1$, where $N_v = N_x \times N_y \times N_z$. The projection matrix encodes the proportion of light contributed by each voxel t in the object volume to each pixel j in the FLFM image. During each iteration, a subset (or patch) of the data is used to update the entire volume.

$$x_j^{k+1} = x_j^k \sum_{t \in S_k} \frac{y_t}{\mu_t^k} H_{tj} \tag{S19}$$

To establish convergence of patch deconvolution to the feasible solution, we aim to prove two propositions (10):

1 The Eqn. S19 converges through iterative updates.

2 The Eqn. S19 converges to the global optimum as Eqn. S18, *i.e.*the residual approaches zero at every pixel between forward-projected image and measured image.

For proving two propositions, three assumptions have to be satisfied:

1 All $H_{tj} \geq 0$, and for each t, there exists at least one $H_{tj} > 0$. This ensures each pixel receives contributions from at least one voxel.

2 Since each patch is treated independently and the PSF represents a probability distribution, it holds that $\sum_{t \in S_i} H_{tj} = 1$ for each subset S_i.

3 The ideal solution (*i.e.*ground truth object) is confined within the FoV of the FLFM; that is, the voxel values outside the FoV are zero.

To prove Proposition 1, assume there exists a strictly positive solution $x^* > 0$ such that $y = Hx^*$ and define $0 * \log 0 = 0$ for blank voxels. Define the Kullback–Leibler (KL) divergence at iteration k as:

$$L^k(x; x^*) = -\sum_{j \in N_v} x_j^* \log(\frac{x_j^*}{x_j^k}) \tag{S20}$$

To show the monotonicity of the KL divergence, the change in KL divergence between iterations can be calculated as (10):

$$
\begin{aligned}
\Delta^k &= L^{k+1} - L^k \\
&= \sum_j x_j^* \log(\frac{x_j^{k+1}}{x_j^k}) \\
&= \sum_j x_j^* \log(\sum_{t\in S_k} \frac{y_t}{\mu_t^k} H_{tj}) \\
&\geq \sum_j x_j^* \sum_{t\in S_k} H_{tj} \log(\frac{y_t}{\mu_t^k}) \\
&= \sum_{t\in S_k} \log(\frac{y_t}{\mu_t^k}) \sum_j H_{tj} x_j^* \\
&= \sum_{t\in S_k} y_t \log(\frac{y_t}{\mu_t^k})
\end{aligned}
$$

where the first inequality follows Jensen's inequality and second inequality follows the inequality $\log x \geq 1 - \frac{1}{x}$. Due to the count preservation in Assumption 2, $\sum_{t\in S_k} y_t = \sum_{t\in S_k} \mu_t^k$, Δ^k can be presented as:

$$
\Delta^k = \sum_{t\in S_k} y_t \log(\frac{y_t}{\mu_t^k}) \geq \sum_{t\in S_k} (y_t - \mu_t^k) = 0 \tag{S21}
$$

To show that L^k is bounded above, we use the inequality again together with the count preservation, leading to $L^k(x; x^*) \leq 0$. Thus, L^k is non-decreasing and bounded, completing the proof of Proposition 1.

For Proposition 2, assume that each patch is selected infinitely often. We first demonstrate that the forward-projected image using a single patch PSF converges to the measured data corresponding to that particular perspective view. Specifically, for $t \in S_1$ (*i.e.*each pixel in the first perspective view), we have $\mu_t^{k_1} \to y_t$ as $k_1 \to \infty$, where $k_1 \in K_1 = \{1, n+1, 2n+1, \ldots\}$ denotes the index sequence corresponding to updates involving the first patch, and n is the total number of patches. According to Proposition 1, where Eqn. S21 tends to zero, such convergence occurs if and only if $\mu_t^{k_1} = y_t$ for each $t \in N_p$. This establishes point-wise convergence of the forward-projected image to the measured image for the selected patch. If S_1 now representing the whole FLFM image, this shows the convergence of RL deconvolution.

The next step is to show $\mu_t^{k_1} \to y_t$ for $k_1 \in K_1$, for $t \in N_p$ (*i.e.*each pixel in the entire FLFM image), which can be written as (10):

$$
\begin{aligned}
&|\sum_j H_{tj} x_j^{k_1} - y_t| \\
&\qquad = |\sum_j H_{tj} x_j^{k_i} \prod_{k=k_i}^{k_1-1} (\sum_{t\in S_k} \frac{y_t}{\mu_t^k} H_{tj}) - y_t| \\
&\qquad = |\sum_j H_{tj} x_j^{k_i} \exp\{\sum_{k=k_i}^{k_1-1} \log(\sum_{t\in S_k} \frac{y_t}{\mu_t^k} H_{tj})\} - y_t| \\
&\qquad \leq |\sum_j H_{tj} x_j^{k_i} - y_t| + |\sum_j H_{tj} x_j^{k_i} \{\exp[\sum_{k=k_i}^{k_1-1} \log(\sum_{t\in S_k} \frac{y_t}{\mu_t^k} H_{tj})] - 1\}|
\end{aligned}
$$

The inequality arises from the application of the triangle inequality. Here, $i \in 2, \ldots, n$ and $k_i \in K_i = \{i, n+i, 2n+i, \ldots\}$ is the largest integer less than k_1, which representing the index corresponding to the completion of a full cycle over all patches prior to iteration k_1.

As $k_i \to \infty$, the first term at RHS tends to zero due to convergence of individual patches. The second term also vanishes because the exponential term converges to 1, as a result of pixel-wise convergence $\mu_k^t \to y_t$, which leads to that $\sum_{t\in S_k} H_{tj} = 1$

(Assumption 2). Thus, the forward projection converges to the measurement globally, completing the proof of Proposition 2. In RL deconvolution, each set S_k represents the entire FLFM image and is identical across iterations. Hence, the same convergence result holds for RL deconvolution.

Under Assumption 3, only the voxels within the FoV contribute to the reconstruction. Consequently, the coordinate mapping from the global coordinate system to the local FoV coordinate system ($s \rightarrow s_p$) does not affect the validity of the convergence proof.

Finally, because the KL divergence $L(x; x^*)$ is strictly convex and continuous, the only accumulation point of the sequence $\{x^k\}$ is the ground truth x^*, completing the proof.

S3 VALIDATION OF PATCH DECONVOLUTION USING SIMULATED DATA

S3.1 Simulation pipeline

To evaluate the performance of patch deconvolution under conditions where the PSF model is known, a pipeline was established to generate simulated data from high-resolution ground truth and perform reconstructions. The pipeline comprises two main components: the simulation and the reconstruction. In the simulation, high-resolution confocal ground truth data (Fig. S4a) were acquired using a confocal microscope, as described in the Methods section. Individual cells were then cropped and downsampled (Fig. S4b) to match the pixel size of the FLFM microscope at the sample plane, also detailed in the Methods section. For reconstruction comparison, the downsampled cells were convolved with the system's PSF (Eqn. S9) to generate simulated FLFM images. Each FLFM image was then reconstructed using both RL deconvolution (Fig. S4c) and patch deconvolution (Fig. S4d). The resulting RL deconvolution volumes were subsequently cropped to match the size of the corresponding patch deconvolution result.

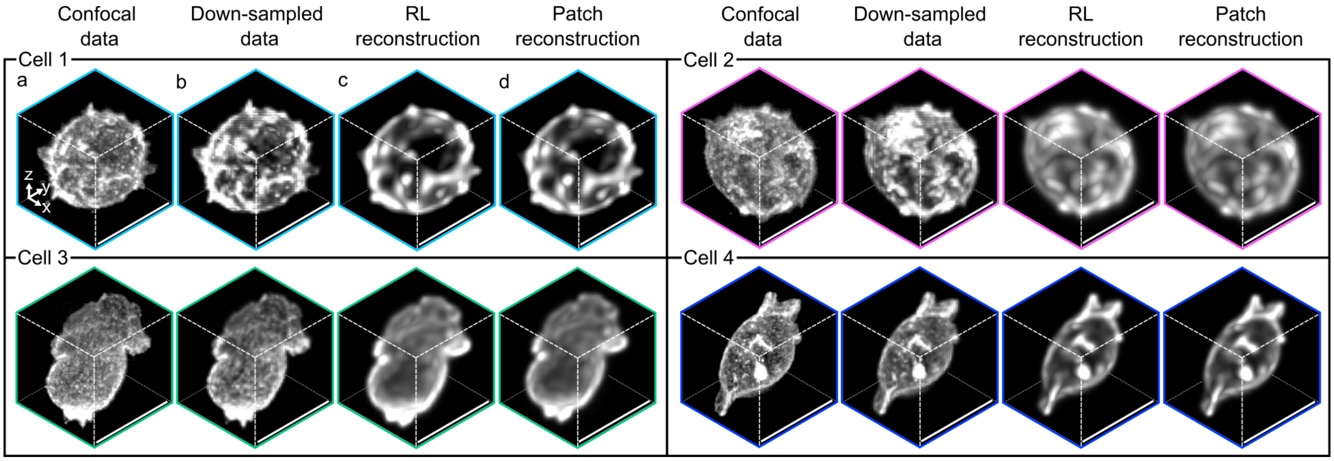

Figure S4: **Simulation pipeline gallery. a** Confocal ground truth data, **b** data down-sampled to match the pixel size of FLFM microscope, **c** RL reconstruction, and **d** patch-based reconstruction. The scale bar represents 15 µm.

S3.2 Simulated Jurkat cell reconstruction

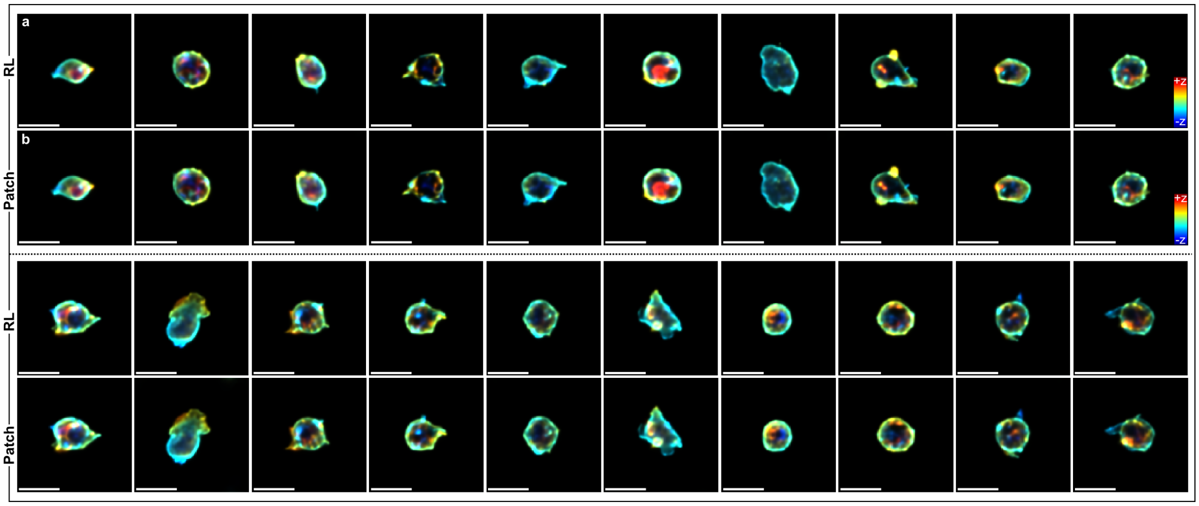

Figure S5: **Simulated Jurkat cell reconstruction gallery.** Maximum intensity projection of cells reconstructed using the RL deconvolution and patch deconvolution, showing various structural features with depth information encoded by colour. The scale bar represents 15 µm.

S3.3 Convergence of deconvolution using more iterations

As RL is a semi-convergence algorithm (6, 10), further iterations beyond the optimum lead to divergence and overfitting. To evaluate the divergence characteristics between RL and patch deconvolution, we extended the number of iterations to 250 on the same set of simulated data, rather than stopping at the number of iterations equal to the number of patches. For this analysis, the downsampled ground truth data (Fig. S4b) were used as the reference instead of selecting the 37th RL iteration as a baseline. Mean squared error (MSE) was then computed by comparing the reconstructed volumes from both RL and patch deconvolution to the downsampled ground truth. Convergence tests were performed under both high PSNR (80 dB) and typical short-exposure (11) imaging conditions (PSNR = 20 dB). At 80 dB PSNR (Fig. S6a), both RL and patch deconvolution exhibited similar convergence rates, with minimal noise and no obvious overfitting. However, at 20 dB PSNR (Fig. S6b), clear divergence occurred after approximately 50 iterations for patch deconvolution and 100 iterations for RL deconvolution. Both methods showed comparable divergence behaviour, with oscillations in the patch deconvolution MSE curve coming from the use of different patches in each iteration. Overall, the convergence and divergence of the two methods were similar.

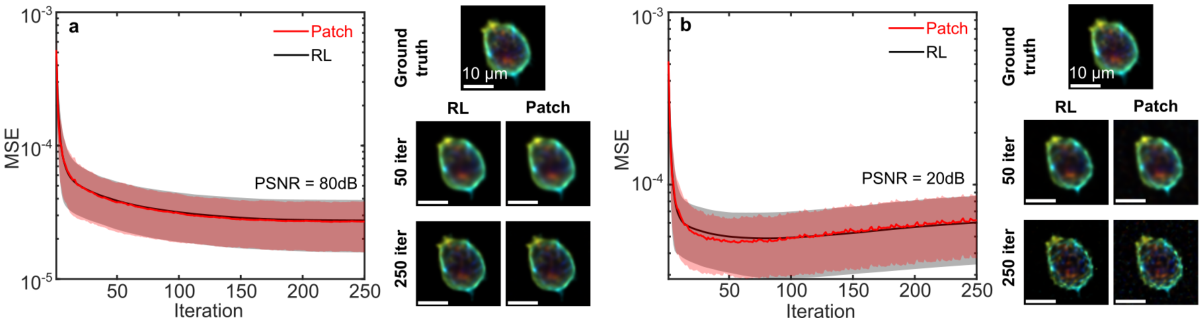

Figure S6: **Convergence comparison between RL deconvolution and patch deconvolution through larger number of iterations using MSE. a** MSE between reconstructed volumes from RL deconvolution and patch deconvolution against simulated cell data over 250 iterations with PSNR = 80 dB. **b** MSE between reconstructed volumes from RL deconvolution and patch deconvolution against simulated cell data over 250 iterations with PSNR = 20 dB, representing typical short-exposure microscopy imaging (11). Maximum intensity projections of a cell, with depth encoded by colour, are shown for both PSNR conditions and both methods. The scale bar represents 15 µm

S4 VALIDATION OF PATCH DECONVOLUTION USING EXPERIMENTAL DATA

S4.1 Bead reconstruction

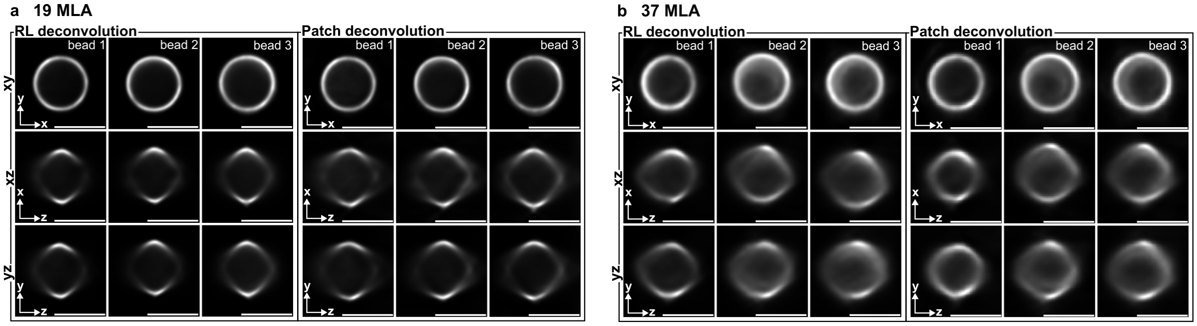

Figure S7: **Experimental bead reconstruction gallery.** Bead reconstructions from RL and patch deconvolution under 19 MLA setup **a** and 37 MLA setup **b** with a 15 µm diameter shown in the xy, xz, and yz planes. The scale bar represents 15 µm.

S4.2 Static Jurkat cell reconstruction

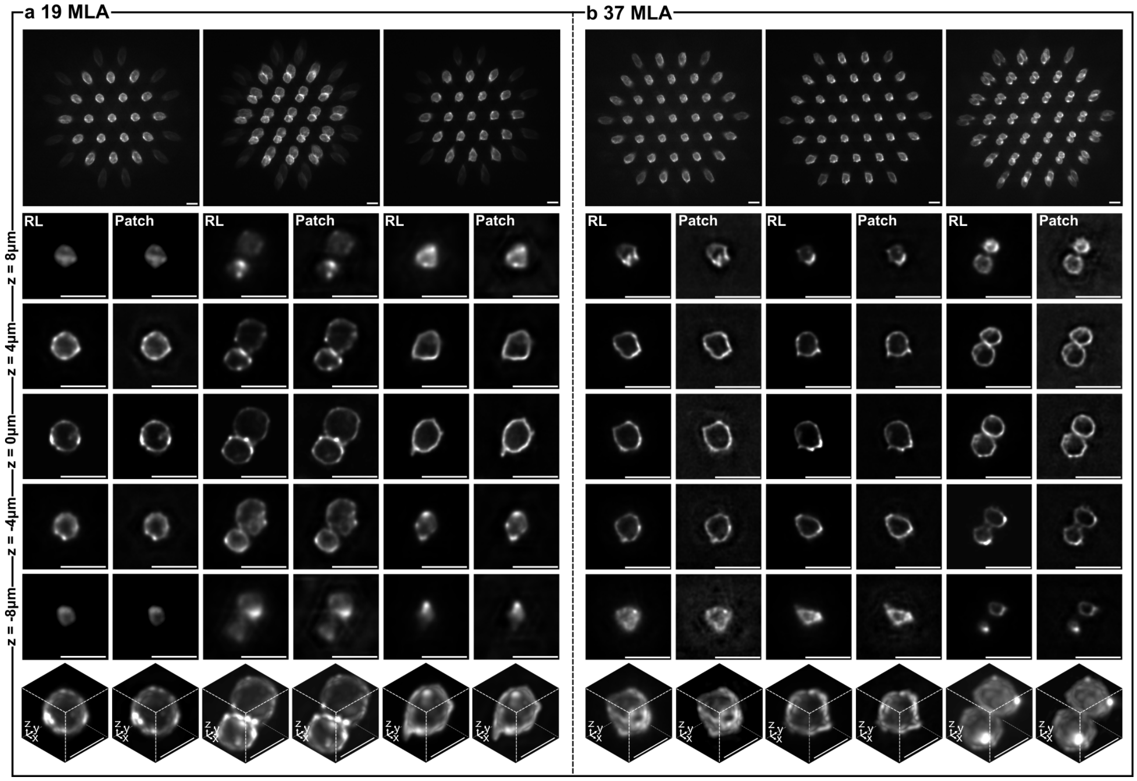

Figure S8: **Static Jurkat cell reconstruction gallery.** Reconstruction of static Jurkat cells using RL and patch deconvolution under the 19 MLA setup **a** and the 37 MLA setup **b**. Axial slices ranging from -8µm to 8 µm are shown alongside the final reconstructed 3D volumes for both methods. The scale bar represents 15 µm.

S4.3 Flow Jurkat cell reconstruction

Besides 0.5 ms exposure time in 16 bit mode discussed in the main text, we also tried 0.1 ms exposure time in 8 bit mode (Fig. S9a), equivalent to 1500 events per second, the fastest frame rate of the camera. If the FoV were cropped to 512 × 512 as stated by Hua *et al.* (5), the similar 5000 events/s acquisition speed could be achieved, which is suitable for sub-cellular imaging. 3D reconstructions remained largely consistent under 0.1 ms exposure time (Fig. S9b), though patch deconvolution exhibited higher background noise, which can be better seen in Fig. S11 from a brighter background contributed by the noise. Nevertheless, such a noise did not heavily influence the the morphology comparison and the following analysis based on the volume (Fig. S9c) even with a lower PSNR (Fig. S9d)

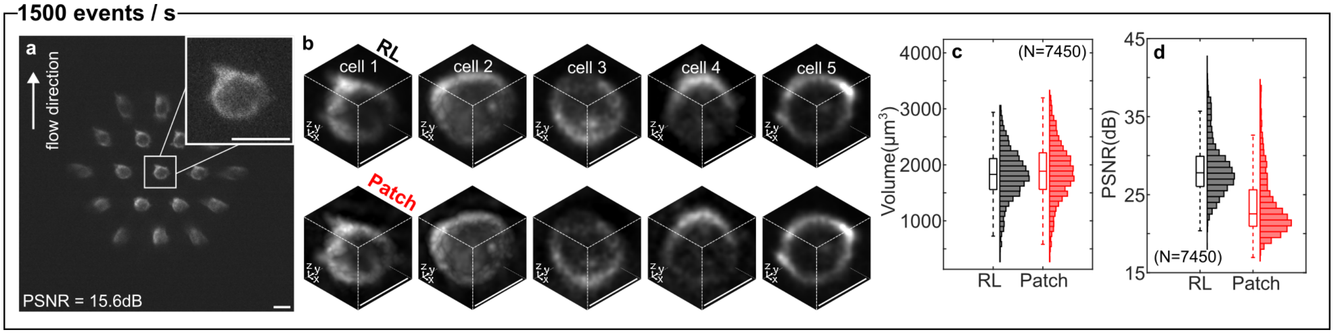

Figure S9: **Validation on flow data with 0.1 ms exposure time. a** The FLFM image with 0.1 ms exposure time from flow experiment. **b** 3D volume renderings of five cells reconstructed using patch and RL deconvolution at 0.1 ms exposure time. **c** Comparison of cell volumes from patch and RL deconvolution. **d** PSNR comparison of reconstructed volumes between patch and RL deconvolution. The scale bar represents 15 µm.

The number of events per second was determined using signals from the Kinetix camera's readout and exposure out ports (12) using a global shutter mode. A BNC cable connected these ports to an oscilloscope, where the readout port indicates digitisation status and the expose out port indicates exposure status of the camera. As shown in Fig. S10a,b, readout from the previous frame ends before the exposure of current frame finishes, and the start of the next exposure is dependent on the camera's readout time. The frame size was set to 1000 × 1000 to capture the full FLFM image for both 19 MLA and 37 MLA configurations. In 16 bit mode, the readout time was 3.54 ms, while in 8 bit mode it was reduced to 0.63 ms.

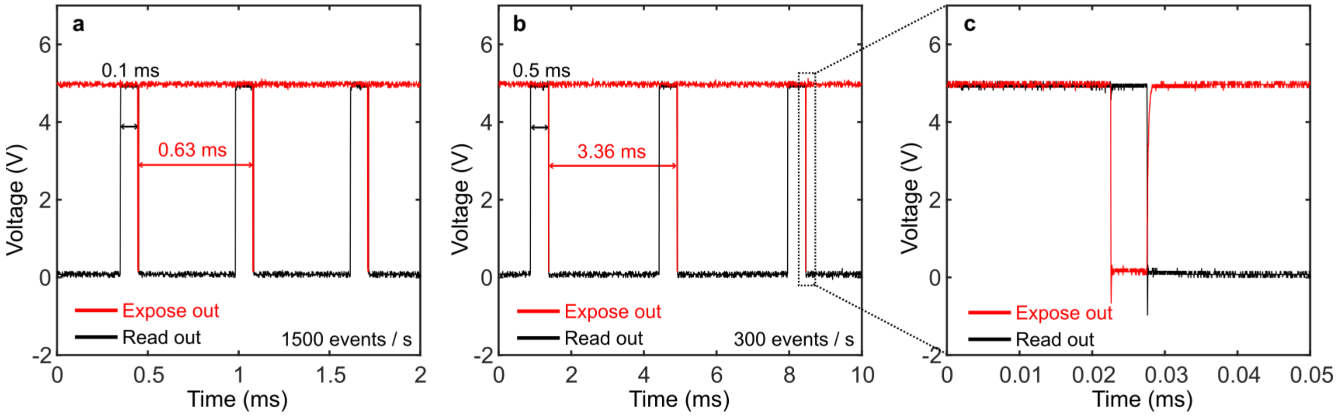

Figure S10: **Voltage output from Kinetix camera in global shutter mode. a** Voltage signals from the expose out and readout ports of the Kinetix camera at 0.1 ms exposure, operating in 8 bit mode with a frame size of 1000×1000, approximately equivalent to 1500 events per second. **b** Same signals at 0.5 ms exposure in 16-bit mode, approximately equivalent to 300 events per second. **c** Zoomed-in view showing the end of the readout and exposure periods. High voltage indicates active operation; low voltage indicates inactivity.

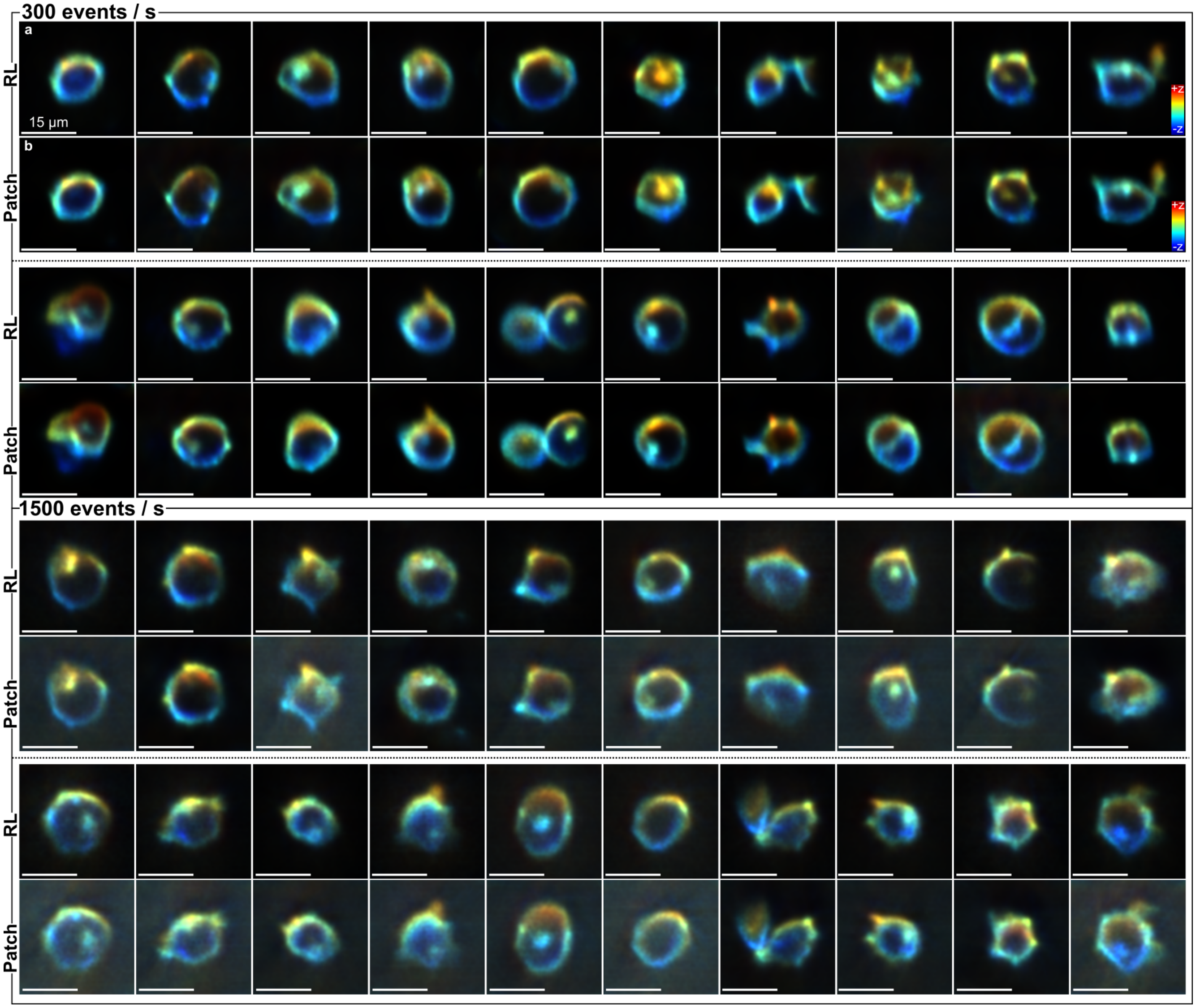

Figure S11: **Flow Jurkat cell reconstruction gallery.** Maximum intensity projection of cells reconstructed using the RL deconvolution and patch deconvolution, showing various structural features with depth information encoded by colour. The scale bar represents 15 µm.

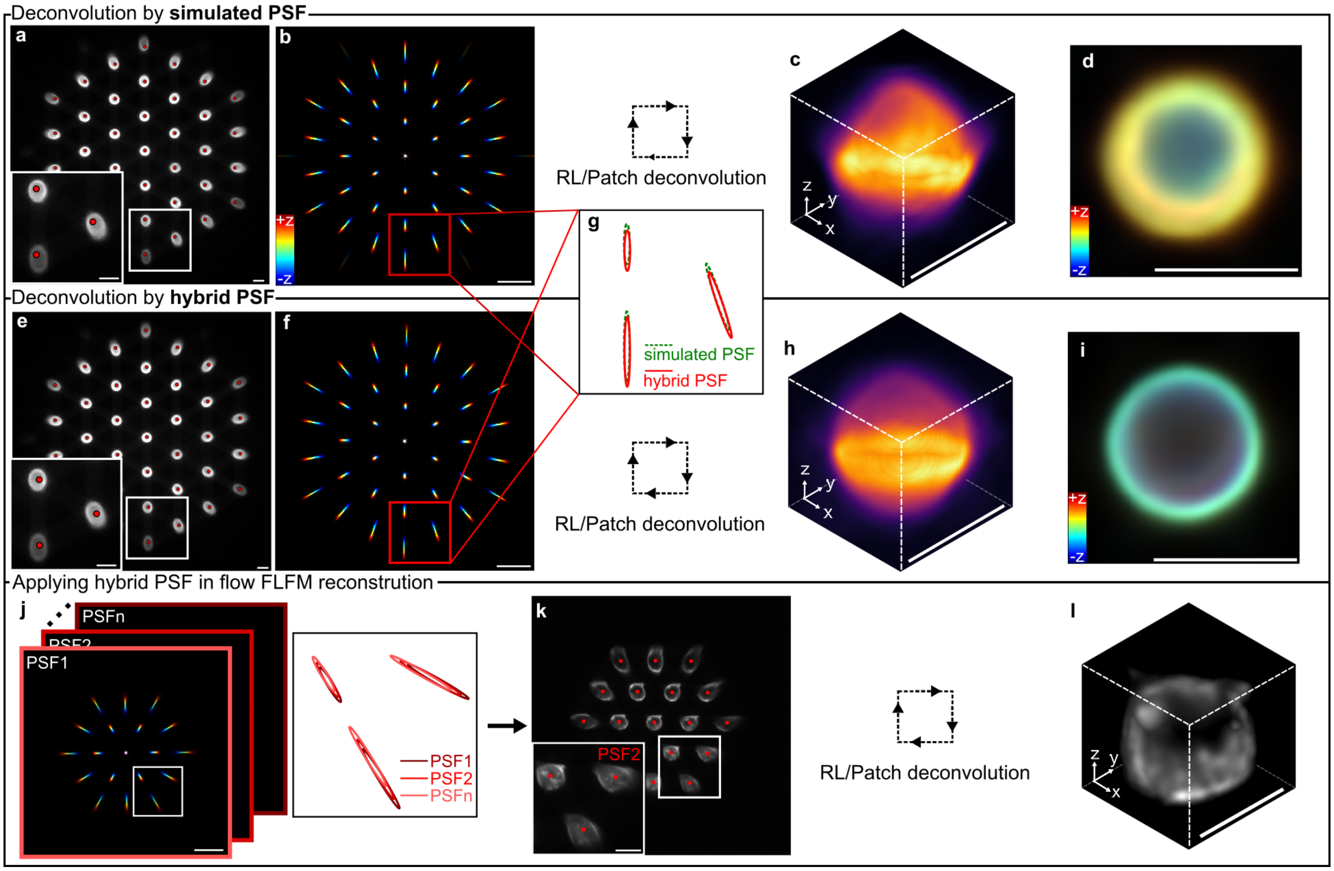

Figure S12: **Comparison between simulated PSF and hybrid PSF. a,e** FLFM image for an experimental bead image with simulated MLA centres **a** and geometric centres **e** of the bead. **b,f** Simulated PSF based on the simulated MLA centres **a** and based on geometric centres **e** (*i.e.*hybrid PSF). **c,h** 3D volumetric reconstruction of a fluorescent object by RL deconvolution using the simulated PSF **b** and hybrid PSF **f**, showing the object structure in x, y, and z dimensions. **d,i** 2D depth color-coded image of the reconstructed object **c,h**, indicating depth variations. **g** Schematic illustration comparing the 3D contour shapes of the simulated (green) and hybrid (red) PSFs, demonstrating the differences in their geometric shape. **j** The pre-simulated PSF library with different distances between views. **k** Selection of PSF from the library based on the current frame. **l** The reconstructed cell from flow using the pre-determined PSF. The scale bar represents 15 µm.

S5 IMPLEMENTATION OF PATCH DECONVOLUTION ON FLOW DATA

Both RL deconvolution and patch deconvolution algorithms, as described in Eqn. S16 and S17, rely on an accurate system PSF for reconstruction. However, various optical aberrations, including defocus from sample drifting within the microfluidic channel and spherical aberration caused by refractive index mismatches between the sample and immersion oil, can lead to discrepancies between the simulated PSF and the actual experimental FLFM data (Fig. S12a). According to Eqn. S9, misalignment between the PSF and the perspectives in the FLFM image can lead to shift of the reconstruction axially (Fig. S12d) and thus reduce the effective DoF of the reconstructed volume. This degrades the overall quality of the reconstruction (Fig. S12c,d).

To address these challenges, we adopted a hybrid PSF approach, combining experimental data into the simulated model. By adjusting the spatial positioning of the PSF based on experimental results, this method compensates for optical aberrations in the simulation model (Fig. S12g), leading to a significant improvement in the reconstruction axial position recovering (Fig. S12i) and thus the reconstruction quality (Fig. S12h). This hybrid PSF approach can produce a better reconstruction quality compared to using experimentally measured PSFs from sub-diffraction beads alone, as it offers a noise-free performance and better PSF sampling due to the higher bit depth of the simulated model.

In the flow experiment, simulating a PSF for each frame is computationally intensive. To address this, a PSF library was pre-generated by varying the pitch of the microlens, which corresponds to changes in the distance between views caused by aberrations (Fig. S12j). For each acquired frame, the average distance between views was computed, and the corresponding pre-generated PSF with the closest matching distance between views was selected for reconstruction (Fig. S12k). As a result, a satisfiable result quality (Fig. S12l) was acquired with no additional time on PSF simulation.

S6 IMPORTANCE OF 3D INFORMATION IN FLOW CYTOMETRY

We conducted a biological experiment using both conventional flow cytometry and 3D fluorescence microscopy to highlight the importance of spatial information in flow cytometry analysis. We carried out a T cell-antigen-presenting cell (APC) coculture assay using the Jurkat T cell line, which is typically evaluated using FACS, here instead focusing on spatial metrics. Jurkat cells expressing the inhibitory receptor PD-1 were cultured with APCs either with or without PD-L1 (the ligand for PD-1) to induce two different levels of activation (13). The expression of PD-1 and CD69, an activation marker, were then analysed on the Jurkat cells (Fig. S13a). While these proteins are known to be expressed at the plasma membrane, spatial information remains unexplored, particularly at the population scale.

Activated Jurkat cells were first analysed using a conventional flow cytometer. After standard gating, the intensity distributions from forward scatter (FSC) and red laser channel (RL1) were obtained (Fig. S13b), revealing two clusters in RL1 consistent with the expected medium and high CD69 expression. The same samples were then imaged using a confocal microscope. Data were down-sampled, transformed into FLFM images, and reconstructed using patch deconvolution (Fig. S4). The mean fluorescence intensity (MFI) was computed by averaging voxel values within the binary mask of each cell, and cell volume was determined based on the number of voxels within the filled cell body mask. Fig. S13c shows a scatter plot of MFI versus volume, revealing two distinct clusters, consistent with results obtained from conventional flow cytometry.

To further leverage 3D spatial information, we calculated the spatial correlation between CD69 and PD-1 distributions (Fig. S14d) on cells with negative PD-L1 binding (*i.e.*high intensity in both PD-1 and CD69 channel), where values approaching 1 indicate strong colocalisation, and values near 0 indicate no spatial association. Most Jurkat cells exhibited strong spatial correlation (>0.9) between CD69 and PD-1 (Fig. S14e). However, cells 1 (blue) and 2 (pink), which had similar volume and intensity values (Fig. S13f) and were therefore indistinguishable in the MFI vs. volume plot (Fig. S13g), showed markedly different spatial correlations (cell 1 = 0.23; cell 2 = 0.97). A gallery of cells with similar MFI values but differing spatial correlation is shown in Fig. S14. This example illustrates the power of 3D spatial information in identifying cellular features that conventional flow cytometry methods overlook. Such capabilities could enable the detection of rare spatial events or distinct subpopulations, providing new dimensions for analysing heterogeneous cellular responses and complex cell behaviours.

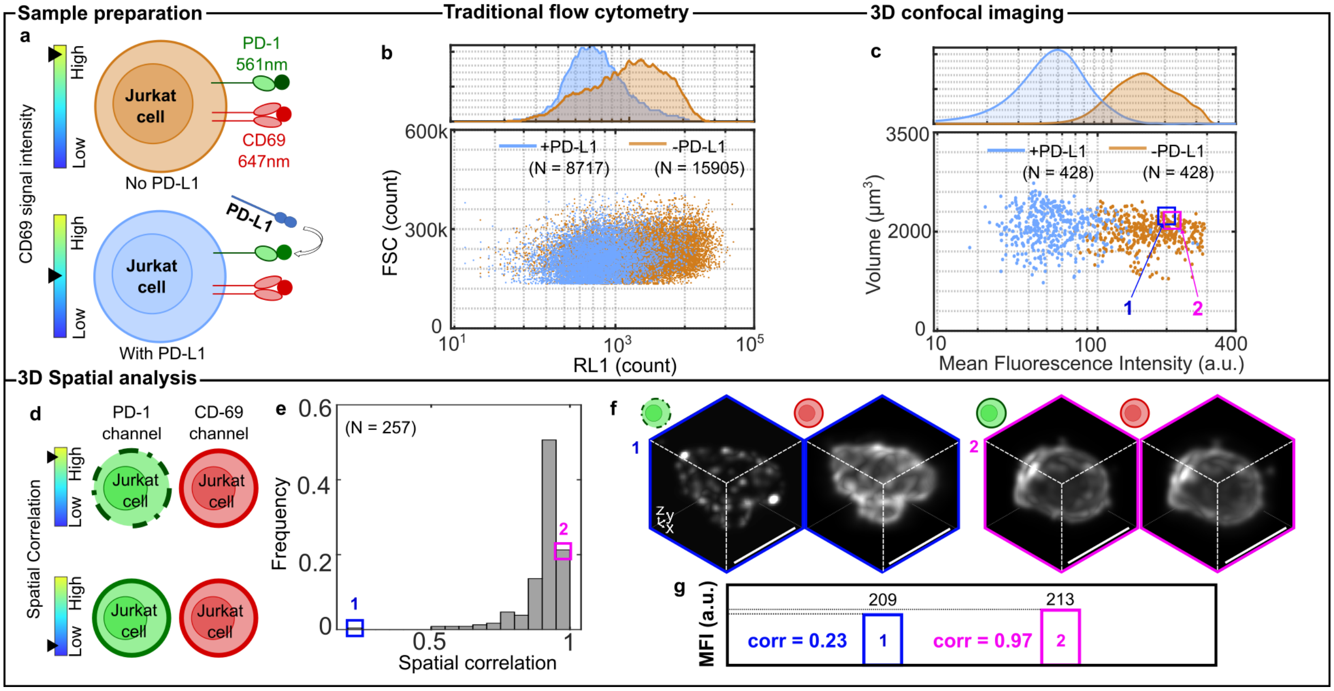

Figure S13: **Importance of 3D information in flow cytometry.** **a** Jurkat cells transduced with PD-1 with and without PD-L1 binding. CD69 expression indicates cell activation level. **b** FSC vs RL1 plot compares PD-L1 positive (orange) and negative (blue) cells after gating. **c** Comparison of cell volume and mean fluorescence intensity (MFI) for +PD-L1 (orange) and -PD-L1 (blue) cells using a confocal microscope. **d** 3D spatial correlation analysis where low spatial correlation indicates different membrane distributions of PD-1 and CD69 on the cell surface. **e** Histogram showing spatial correlation results for Jurkat cells without PD-L1 binding and with signals in both CD69 and PD-1 channels. **f** Cell (1) shows low spatial correlation (corr = 0.23) while cell (2) shows high spatial correlation (corr = 0.97). **g** Box plots showing MFI values for PD-1 and CD69 for the two example cells. The scale bar represents 15 μm.

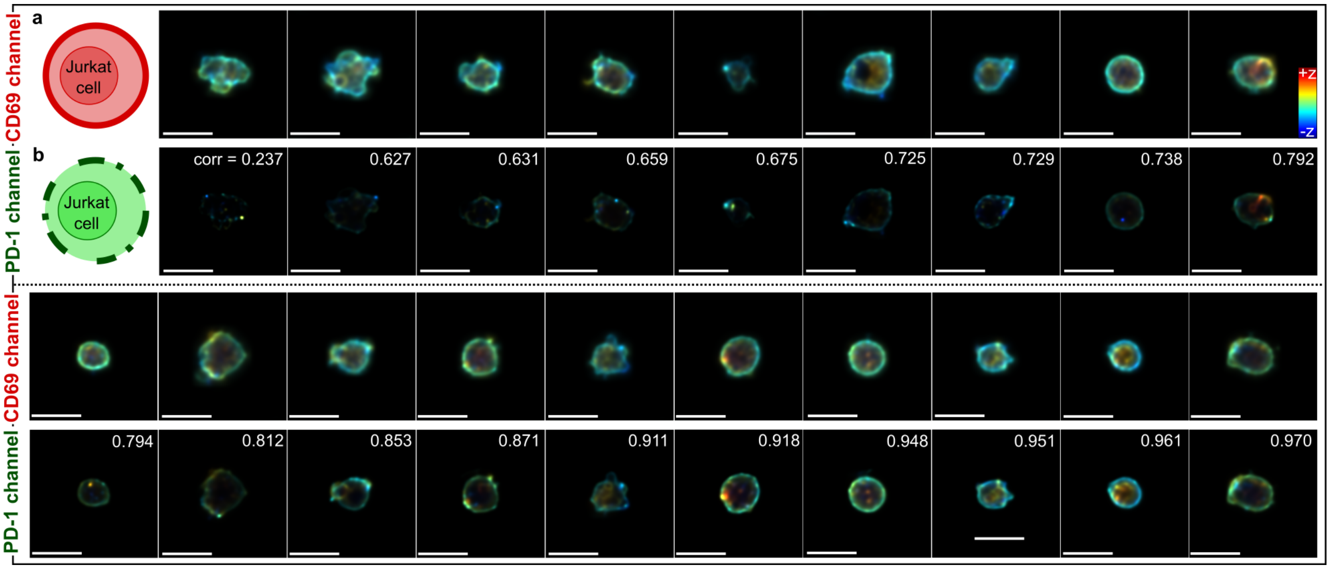

Figure S14: **CD69 and PD-1 spatial correlation gallery.** Maximum intensity projection of reconstructed volume in CD69 channel (**a**, top row) and PD-1 channel (**b**, bottom row) with depth information encoded by colour. The scale bar represents 15 μm.

REFERENCES

1. Guo, C., W. Liu, X. Hua, H. Li, and S. Jia, 2019. Fourier light-field microscopy. *Optics Express* 27:25573.
2. Galdón, L., G. Saavedra, J. Garcia-Sucerquia, M. Martínez-Corral, and E. Sánchez-Ortiga, 2022. Fourier lightfield microscopy: a practical design guide. *Applied Optics* 61:2558.
3. Sims, R. R., S. A. Rehman, M. O. Lenz, S. I. Benaissa, E. Bruggeman, A. Clark, E. W. Sanders, A. Ponjavic, L. Muresan, S. F. Lee, and K. O'Holleran, 2020. Single molecule light field microscopy. *Optica* 7:1065.
4. Born, M., E. Wolf, A. B. Bhatia, P. C. Clemmow, D. Gabor, A. R. Stokes, A. M. Taylor, P. A. Wayman, and W. L. Wilcock, 1999. Principles of Optics: Electromagnetic Theory of Propagation, Interference and Diffraction of Light. Cambridge University Press, 7 edition.
5. Hua, X., K. Han, B. Mandracchia, A. Radmand, W. Liu, H. Kim, Z. Yuan, S. M. Ehrlich, K. Li, C. Zheng, J. Son, A. D. S. Trenkle, G. A. Kwong, C. Zhu, J. E. Dahlman, and S. Jia, 2024. Light-field flow cytometry for high-resolution, volumetric and multiparametric 3D single-cell analysis. *Nature Communications* 15.
6. Dey, N., L. Blanc-Feraud, C. Zimmer, P. Roux, Z. Kam, J. C. Olivo-Marin, and J. Zerubia, 2006. Richardson-Lucy algorithm with total variation regularization for 3D confocal microscope deconvolution. *Microscopy Research and Technique* 69:260–266.
7. Richardson, W. H., 1972. Bayesian-Based Iterative Method of Image Restoration. *JOURNAL OF THE OPTICAL SOCIETY OF AMERICA* 62:55–59.
8. Lucy, L. B., 1974. An iterative technique for the rectification of observed distributions. *THE ASTRONOMICAL JOURNAL* 79:745–754.
9. Biggs, D. S. C., and M. Andrews, 1997. Acceleration of iterative image restoration algorithms. *Applied Optics* 36:1766–1775.
10. Hudson, H. M., and R. S. Larkin, 1994. Accelerated Image Reconstruction Using Ordered Subsets of Projection Data. *IEEE TRANSACTIONS ON MEDICAL IMAGING* 13:601–609.
11. Chen, F., J. Liu, D. Gou, X. Zhang, L. Chen, and H. Liao, 2020. An accurate and universal approach for short-exposure-time microscopy image enhancement. *Computerized Medical Imaging and Graphics* 83.
12. Teledyne Photometrics, 2021. Kinetix Camera Manual. Teledyne Vision Solutions / Teledyne Photometrics. https://www.photometrics.com/wp-content/uploads/2021/11/Kinetix-Manual-58-723-004_RevA01.pdf, rev. A01; PDF available online.
13. Patsoukis, N., Q. Wang, L. Strauss, and V. A. Boussiotis, 2020. Revisiting the PD-1 pathway. *Sci. Adv* 6.
