# Supplementary material for: Patch deconvolution for Fourier light-field microscopy
Source: Biophys J. 2026 Jan 21;125(5):1305–14. doi: 10.1016/j.bpj.2026.01.034 (PMC13351718; doi:10.1016/j.bpj.2026.01.034)
Supplement: Document S2. Article plus Supporting Material [file mmc3.pdf]

Biophysical Journal
Article

Biophysical Society

Patch deconvolution for Fourier light-field microscopy

Bin Fu,[1] Caroline L. Jones,[1] Daniel Heraghty,[1] Shengbo Yang,[1] Caitlin O'Brien-Ball,[2] Victoria Junghans,[2] Haowei Yang,[1] David Klenerman,[1] Tuomas P. J. Knowles,[1] Lucien E. Weiss,[3] Ricardo A. Fernandes,[2] and Steven F. Lee[1,*]

[1]Yusuf Hamied Department of Chemistry, University of Cambridge, Lensfield Road, Cambridge, UK; [2]Chinese Academy of Medical Sciences (CAMS) Oxford Institute (COI), University of Oxford, Oxford, UK; and [3]Department of Engineering Physics, Polytechnique Montréal, Montréal, Québec, Canada

ABSTRACT Imaging flow cytometry using Fourier light-field microscopy enables high-throughput three-dimensional cellular imaging, capable of capturing thousands of events per second. However, volumetric reconstruction speed remains orders of magnitude slower than the acquisition speed. The current state of art uses Richardson-Lucy algorithm, restricted to just 5–10 reconstructed events per second with GPU acceleration. This limitation hinders real-time applications such as cell sorting and thus has bottlenecked the widespread adoption of 3D imaging flow cytometry. We introduce patch deconvolution, the first training-free algorithm compatible with the Richardson-Lucy framework that significantly accelerates convergence, achieving over 100–200 reconstructions per second on standard GPUs, a 20- to 40-fold improvement over Richardson-Lucy. Validated on both simulated and experimental data sets, patch deconvolution achieves reconstruction quality comparable to Richardson-Lucy in both static and flow data. This supports rapid cell sorting based on spatial features and enables advanced applications, such as detecting rare spatial events in large cell populations, which would otherwise be indistinguishable in traditional flow cytometry.

SIGNIFICANCE This project significantly advances 3D imaging flow cytometry by introducing patch deconvolution, a technique that accelerates volumetric reconstruction speeds by 20–40 times over the traditional Richardson-Lucy algorithm. Compatible with current software framework, it achieves over 100–200 reconstructions per second without sacrificing image quality. This breakthrough overcomes a major bottleneck in real-time applications such as cell sorting and enables the detection of rare spatial events in large cell populations. By bridging the gap between acquisition and reconstruction speeds, the method enhances the practicality and adoption of high-throughput, 3D cellular imaging in biomedical research and diagnostics.

INTRODUCTION

Over the past 60 years, flow cytometry has become an indispensable tool for cell biologists to interrogate population-scale data (1). By measuring the light scattered and emitted as cells pass through a focused light beam, flow cytometers along with additional hardware and control systems can also sort cells quickly. For instance, fluorescence-activated cell sorting enables high-throughput sorting of thousands of cells per second but is limited to low-resolution parameter spaces (Fig. 1 *a*), e.g., intensity and size (1). Alternatively, fluorescence microscopy enables detailed spatial information but generally has low throughput. Imaging flow cytometry (IFC) combines the strengths of both techniques, merging the speed of flow cytometry with the spatial detail of microscopy (2–4). As such, IFC allows investigation of population-level dynamics based on complex spatial metrics of individual cells such as eccentricity, spatial clustering, and correlation between fluorescent labels (Fig. 1 *a*) (5,6). IFC, such as developed by Schraivogel et al.(7), achieves cell sorting at rates of 15,000 events/s using a low-latency image-processing algorithm to make real-time, image-based sorting decisions. However, IFC approaches rely on 2D images of cells, limiting their ability to capture the full 3D spatial information, which is often critical for understanding cellular

Submitted August 7, 2025, and accepted for publication January 16, 2026.

*Correspondence: sl591@cam.ac.uk

Editor Name: Maria Garcia-Parajo

https://doi.org/10.1016/j.bpj.2026.01.034

© 2026 The Author(s). Published by Elsevier Inc. on behalf of Biophysical Society.
This is an open access article under the CC BY license (http://creativecommons.org/licenses/by/4.0/).

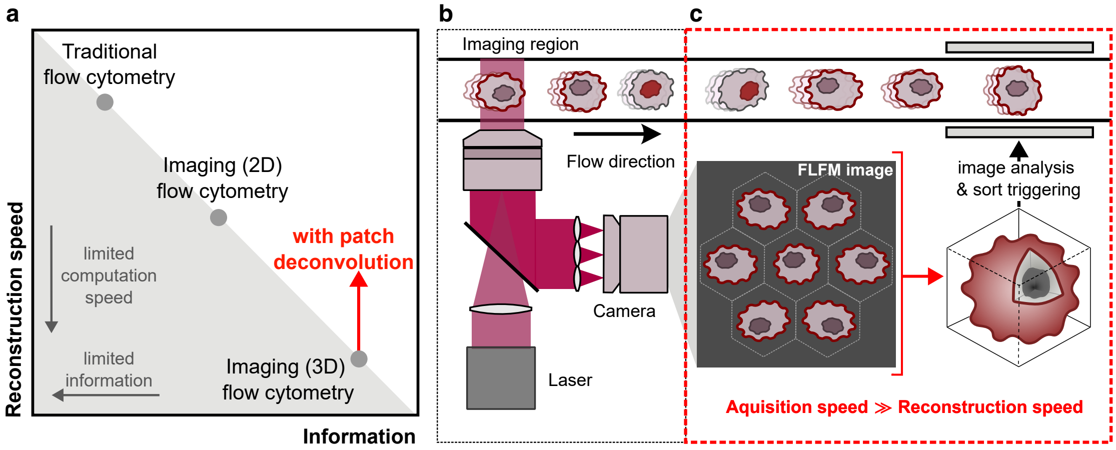

FIGURE 1 Patch deconvolution enables cell sorting for 3D imaging flow cytometry. (*a*) Comparison of traditional flow cytometry, 2D imaging flow cytometry, and 3D imaging flow cytometry in terms of reconstruction speed and information content. The bottleneck in 3D imaging cytometry arises from the slow reconstruction speeds. Patch deconvolution (*red arrow*) is proposed to address this, enabling faster reconstruction speed for 3D flow cytometry. (*b*) Schematic of a 3D flow cytometry setup using a high NA objective, laser illumination, and a microlens array (MLA) to capture a FLFM image for flowing cells. (*c*) Workflow of FLFM image acquisition and image reconstruction for sorting. Patch deconvolution accelerates the time-intensive image reconstruction step.

structure and function in biological systems (3), such as protein traffics (8) and protein-protein colocalizations (9).

Efforts to extend flow cytometry to high-throughput 3D imaging have shown promise but face significant trade-offs, including reduced throughput (<100 events per second) (10,11), increased system complexity (12–14), or limited depth of field and emitter density (15). Light-field flow cytometry (LFC), the implementation using Fourier light-field microscopy (FLFM) (16–19) as demonstrated by Hua and Han et al., addresses these challenges. FLFM captures multiple perspectives of the sample in a single snapshot, achieving throughputs of up to 5000 events/s on a 512 × 512 image (Fig. 1 *b*) (20). However, accurate reconstruction relies heavily on computationally intensive deconvolution algorithms. Richardson-Lucy (RL) deconvolution (21,22), an iterative expectation-maximization (EM) algorithm (23,24), is widely used due to its robustness and simplicity (25). In the system described by Hua and Han et al., a 4-μm depth of field (DOF) requires approximately 10 s per volume for RL deconvolution. To capture entire cells, a greater number of microlenses is required for a larger DOF, resulting in a larger image and volume size and a corresponding quadratic increase in reconstruction time. This creates a bottleneck, where reconstruction times, often taking days for large data sets, far exceed imaging times, thus limiting LFC's practical use in real-time applications such as cell sorting (Fig. 1 *c*).

Various strategies have been explored to accelerate RL deconvolution, including algorithmic optimizations, deep learning approaches, and hardware-efficient matrix optimizations. Algorithmic methods focus on accelerating convergence by modifying the objective function (26–29), such as incorporating Wiener-Butterworth filters (28), which can reduce the number of iterations to fewer than five. Nonetheless, even a single iteration typically requires tens of milliseconds and requires a large GPU memory, making it impractical for high-throughput applications, and also, it was proved to be less effective in FLFM reconstruction (29). Deep learning-based reconstruction methods (30–33) also have their own limitations. Aside from the challenge of obtaining large, high-quality ground truth data sets for training, these models often exhibit poor generalizability across varying sample structures, noise levels, and optical aberrations, as demonstrated by Lu et al. (33). Although SeReNet (33), a self-supervised architecture, demonstrates improved generalizability, it still requires hundreds of milliseconds per volume reconstruction. Furthermore, deep learning-based reconstruction models are not universally applicable across different optical configurations, input dimensions, or aberration and noise conditions introduced by new samples. In contrast, deconvolution algorithms remain robust, training free, and compatible with a broad range of system parameters (34). Additionally, matrix-level optimizations (35–37), such as reformulating the RL update step to use more efficient matrix structures (37), offer gains in speed and memory efficiency, particularly on GPU architectures, yet reconstruction times still require tens of iterations to converge and thus remain in the range of hundreds of milliseconds. The comparisons are summarized in Table 1.

To further reduce reconstruction time to the millisecond scale while preserving the advantages of existing optimization strategies, we introduce patch deconvolution, a technique that does not require pretraining and is fully compatible with conventional RL deconvolution frameworks and for significantly improving computational efficiency without compromising reconstruction accuracy. Inspired from Bayesian-based multiview deconvolution

TABLE 1 Comparison of Different Reconstruction Methods

Method	Model	Generalizability	Convergence Speed	Hardware Requirement	Ref
Subset RL with plane-wise optimization	this work	high	$\leq$1 iteration[a]	CPU/GPU	–
Joint RL	Hua et al., Ingaramo et al.	high	20-40 iterations	CPU/GPU	Hua et al., Ingaramo et al.(20,38)
Joint RL with Wiener-Butterworth filter	Guo et al.	high	1–5 iterations	CPU/GPU	Guo et al.(28)
Joint RL with projection-estimation	Wu et al.	high	1–5 iterations	CPU/GPU	Wu et al.(29)
Subset RL	Preibisch et al.	high	$\leq$1 iteration[a]	CPU/GPU	Preibisch et al.(39)
VCD/F-VCD-Net	Wang et al., Yi et al.	low	N/A	GPU	Wang et al., Yi et al.(30,31)
HyLFM-Net	Wagner et al.	low	N/A	GPU	Wagner et al.(32)
SeReNet	Lu et al.	high	N/A	GPU	Lu et al.(33)

The PSF of a FLFM is composed of PSFs from multiple microlenses, analogous in concept to joint RL deconvolution. Therefore, in this table, RL deconvolution applied to FLFM is referred to as joint RL deconvolution.

[a]Here, one iteration denotes a full update using the complete PSF, different from iterations defined previously in the text. The number of iterations for previously reported methods was taken directly from the respective publications. For this work, convergence was empirically defined as the condition under which, after all views were processed once, the relative change in mean squared error (MSE) was less than 5%. Therefore, we report a convergence speed of $\leq$1 iteration for this method. A more rigorous, quantitative convergence criterion could be implemented for heterogeneous data sets (34).

in light-sheet microscopy (39) and ordered-subsets EM (OSEM) in computed tomography (40), patch deconvolution reformulates the update process by sequentially using individual perspective views, rather than processing the entire FLFM image at once, during each iteration. This strategy enables multiple volume updates per point-spread function (PSF), proportional to the number of views captured in the FLFM system. In contrast, standard RL deconvolution performs a single update per iteration using the full PSF. Consequently, patch deconvolution achieves a speedup factor theoretically equal to the number of views, which at least has to be 19 for a whole-cell imaging (Note S1.1), allowing complete 3D reconstructions to be performed in milliseconds on a GPU. Furthermore, because the method is grounded in the RL framework, it remains fully compatible with existing algorithm-level and matrix-level optimizations and also regularizations (41,42).

In this work, we describe the principle of patch deconvolution and its implementation within existing RL framework. We validate its performance through simulations and experimental data sets (both static and in-flow FLFM at acquisition rates of 300–1500 events/s). Our results demonstrate strong agreement between patch deconvolution and conventional RL reconstructions, both qualitatively and quantitatively. Furthermore, we provide a mathematical proof that, under noise-free and aberration-free conditions, patch deconvolution converges to the same ground truth solution as standard RL deconvolution (Note S2.4). Critically, patch deconvolution enables, for the first time to our knowledge, 3D reconstruction in a millisecond scale for LFC, thereby supporting the highest cell-sorting throughput currently achievable based on 3D data.

Principle of patch deconvolution

RL deconvolution, derived from Bayes' theorem and implemented as an iterative expectation-maximization (EM) algorithm, has been widely employed in astronomy and medical imaging for image restoration due to its simplicity and efficacy (42). Given the PSF of a system, $h(s)$, and the observed image, $I(s)$, where s denotes each pixel, RL deconvolution iteratively estimates the latent image by maximizing the likelihood of the estimated data based on the measured data. Each iteration comprises four sequential steps: forward projection (FP)—convolving the current estimate, $o_k(s)$, with k representing the iteration, with the PSF, $h(s)$, to simulate the measured image; error correction (EC)—the ratio between the measured image, $I(s)$, and the FP result; back projection (BP)—convolving the EC result with the flipped PSF $h(-s)$; and the result update (RU)—multiplying the BP result with the current estimate for updating (Fig. 2 *a*). For implementation of RL deconvolution in FLFM (Fig. 2 *b*), the BP step is represented in Eq. 1 and RU step in Eq. 2, where $f_{RL}(s;z)$ denotes the back-projected image at a specific axial plane, and the integral characterizes the projection of the 3D volume into a 2D image (43).

$$f_{RL}(s;z) = \left[\frac{I(s)}{\int_z o_k(s;z) * h(s;z)dz}\right] * h(-s;z) \quad (1)$$

$$o_{k+1}(s;z) = o_k(s;z) f_{RL}(s;z) \quad (2)$$

Building on the RL deconvolution framework, we introduce the patch deconvolution that takes the advantage of the multiview nature of FLFM, similar to approaches in multiview light-sheet microscopy and computed tomography. In FLFM, a single image comprises multiple perspectives of the same object, resulting from the microlens array (MLA) located in the BFP. Each of these perspective views, or "patches," is effectively independent and associated with its own unique PSF. Consequently, each patch can be deconvolved individually. If an FLFM image contains p perspectives, it can be partitioned into p patches,

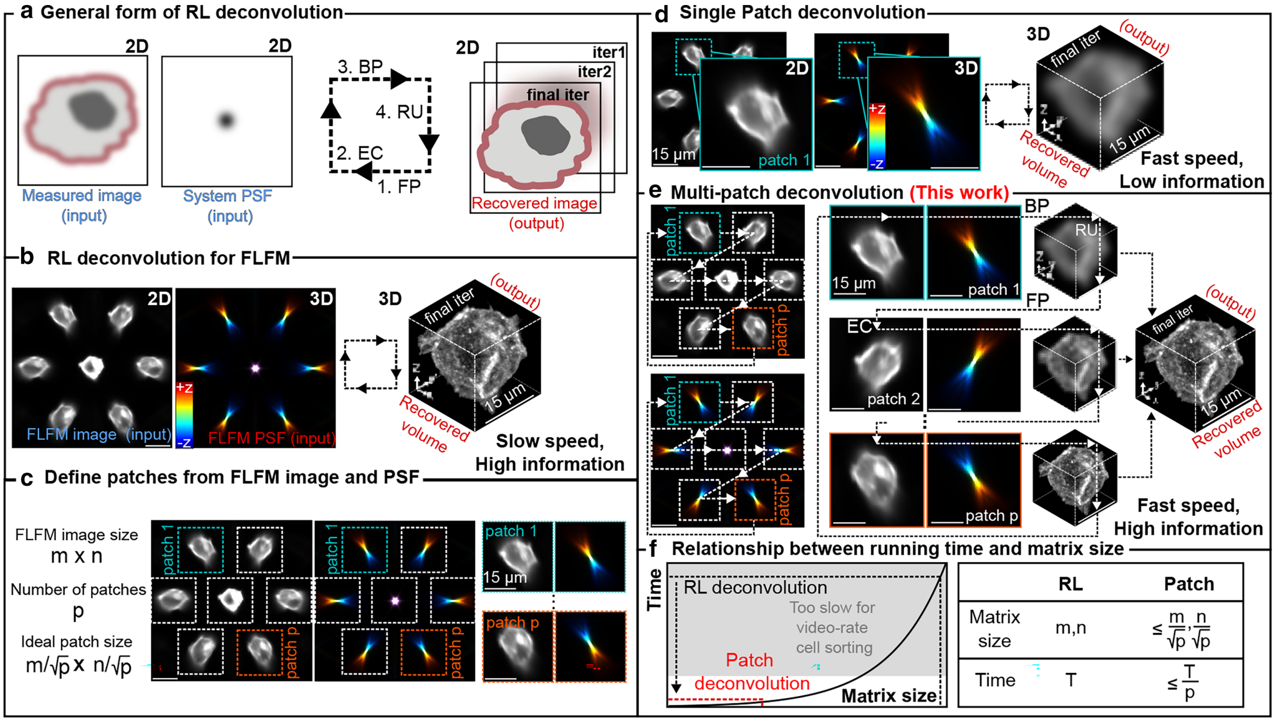

FIGURE 2 RL deconvolution and patch deconvolution for FLFM reconstruction. (*a*) RL deconvolution involves iterative forward projection (FP), error correction (EC), back projection (BP), and result update (RU). (*b*) In FLFM, 3D volumes are reconstructed iteratively from the measured image and PSF but are computationally slow. (*c*) Dividing images and PSFs into patches reduces computation; optimal patch size is $m/\sqrt{p} \times n/\sqrt{p}$. (*d*) Single-patch deconvolution is fast but with low result quality. (*e*) Multipatch deconvolution (also referred to as patched deconvolution in this paper) method. The FLFM image and PSF are divided into multiple patches, with deconvolution performed independently on each patch in sequence. (*f*) Reconstruction runtime scales quadratically with matrix size. Therefore, patching can significantly reduce time.

each ideally of size $m/\sqrt{p} \times n/\sqrt{p}$, where $(m \times n)$ denotes the dimensions of the original image (Fig. 2 *c*).

In patch deconvolution (Fig. 2 *e*), the BP step from a single patch is described in Eq. 3, and the RU step sequentially using views to update reconstructed volume is formalized in Eq. 4. Here, one iteration is defined as processing one patch, with P representing the full set of patches and p a specific patch. For implementation in FLFM, we adopt a plane-wise optimization approach (37). Instead of performing a full 3D deconvolution by padding the 2D FLFM data into a 3D volume, we apply 2D deconvolution using a 2D PSF at each axial plane. This approach enables recovery of 2D volume slices corresponding to each plane, significantly reducing the memory requirements on the GPU.

$$f_{\mathrm{RL}}^{p}(s_p;z) = \left[\frac{I_p(s_p)}{\int_z o_k(s_p;z) * h_p(s_p;z)\,dz}\right] * h(-s_p;z) \quad (3)$$

$$o_{k+p}(s_p;z) = o_k(s_p;z)\prod_{p\in P} f_{\mathrm{RL}}^{p}(s_p;z) \quad (4)$$

The mathematical convergence of patch deconvolution to the global minimum as RL deconvolution has been proved in Note S2.4.

MATERIALS AND METHODS

Experimental setup

Experiments were conducted using one of two distinct instruments: a widefield fluorescence microscope with a MLA for FLFM imaging and a spinning-disk confocal microscope detailed in (44) for acquiring high-resolution data for simulation.

The FLFM microscope was a bespoke widefield fluorescence microscope, with the illumination entering the microscope body through the back illumination port. The excitation path has a 638-nm laser (Odic force lasers, DM-RL500). The laser beam was circularly polarized using quarter-wave plates. Light is then focused onto the back focal plane of an oil immersion objective (Olympus, UPlanSApo 60×/1.30 NA silicon) via a dichroic mirror (Semrock, Di01-R405/488/561/635). Fluorescence emitted from the sample is filtered by an emission filter (Semrock, FF01-432/515/595/730) before passing through a 100-mm or 125-mm Fourier lens (Thorlabs AC254-100-A-ML and AC254-125-A-ML for 19 and 37 MLA configurations, respectively), positioned at a focal length from the native image plane. An MLA, pitch 1 mm and focal length 36.7 mm (Okotech, APH-Q-P1000-F36,7), is placed at a focal length from the Fourier lens. A high-speed sCMOS camera (Photometrics, Kinetix) is positioned at the MLA image plane.

A custom flow-focusing microfluidic device was used in this work. It was fabricated from PDMS on glass coverslips using established protocols (45). An ElveFlow OB1 microfluidic flow controller was used for individual control of the separate sheath and sample channels. A Thorlabs air compressor (Thorlabs, PTA522) was used to pressurize the system. Sheath fluid (PBS) and sample were kept in sealed Eppendorf and delivered independently to the microfluidic device by 1.6-mm OD PTFE tubing (BL-PTFE-1608-20, Darwin Microfluidics) with a 20-mbar pressure. Cells were resuspended

in 1 mL of PBS, which was plumbed into the microfluidic system alongside a separate 1 mL Eppendorf tube of PBS for the sheath channel.

Light-field image formation

The 3D PSF of the FLFM microscope was simulated using formulas described in Note S2.1. The PSF voxel size was set to be the same as the camera pixel size at the object plane across a 20- or 30-μm axial range for the 19 MLA and 37 MLA configurations, respectively. To ensure a full sampling of PSF, the simulation was performed over a matrix larger than the BFP, using a size of 1000 × 1000 pixels.

The ground truth objects were 3D cell volumes cropped and resampled from confocal microscopy data. These volumes were zero-padded in all three dimensions to match the simulated PSF matrix dimensions. The simulated FLFM image was then generated using Eq. 5, where I denotes the simulated FLFM image. These images were normalized to a range between 0 and 1. FLFM images with different levels of noise were then created by adding a series of Poisson noise images with varying standard deviation (σ) to the original noiseless image. The peak signal/noise ratio (PSNR) of the resulting FLFM images was calculated as $20\log_{10}\left(\frac{1}{\sigma}\right)$.

$$I(s) = \int_z o(s;z) * h(s;z)dz \tag{5}$$

Reconstruction for static and flow data

The reconstruction was performed using a self-implemented version of RL deconvolution and patch deconvolution. The number of iterations in the RL deconvolution was set equal to the number of patches used in the patch deconvolution, which was equal to the number of views. All reconstructions were performed using MATLAB 2024a on a PC with an Intel i7-13700K CPU and Nvidia RTX 4060 GPU. For RL deconvolution, the reconstructed volume had the same matrix dimensions as the simulated PSF. In the case of patch deconvolution, the axial dimension of both the volume and PSF matched that used in RL deconvolution. However, the lateral dimensions were limited to 130 × 130 pixels, corresponding to field-of-view (FOV) size in pixel. After reconstruction, the volume obtained from the RL deconvolution was cropped from the center to match the size of the volume reconstructed from the patch deconvolution. Both RL and patch volumes were normalized between 0 and 1. For experimental data sets, a hybrid PSF (Note S5) was used during the reconstruction process.

Sample preparation

Jurkat cells were used in this work for performance comparison. Membrane receptor CD45 on the surface of Jurkat cells was labeled with antibody gap 8.3 and AlexaFluor 647 (concentration 15 μg/mL). Cells were cultured in RPMI-1640 media (Merck/Sigma-Aldrich Cat No: R2405-500ML) with 10% FBS (Thermo Fisher REF:10,500-064), 1% sodium pyruvate (Thermo Fisher REF:11,360-039), 1% HEPES (Thermo Fisher REF:15,630-056), and 1% penicillin-streptomycin (Thermo Fisher REF: 15,140,122). After three washes in 20-nm filtered PBS, cells were suspended in 30 μL of the antibody solution and incubated for 15 min. Cells were then washed twice more in PBS before fixation in 0.8% PFA (thermo scientific, 28,906) and 0.2% glutaraldehyde (Sigma-Aldrich, 3802-75ML) solution. They were left to fix at room temperature for 15 min. Cells were washed two final times before resuspension in PBS for imaging. Cells were plated in a μ-Slide 18-Well Glass Bottom slide (ibidi, 80,807).

RESULT

Validation on static bead and cell data

To evaluate the performance of patch deconvolution, we first tested it on simulated data without noise and aberrations (Fig. 3 *a* and Note S3). Under these conditions, patch deconvolution converged to a result nearly identical to that of RL deconvolution when using the number of iterations equal to the number of views in the FLFM image (i.e., each patch was used once).

We next validated the patch deconvolution on experimental data, imaging static beads and membrane-labeled T-cells using the FLFM microscope with a 100-ms exposure. For bead imaging, fluorescently surface-labeled 15-μm beads (Thermo Fisher, FocalCheck Fluorescence Microscope Test Slide #1) were imaged in both 19 MLA and 37 MLA configurations. Reconstructions were performed using a hybrid PSF that accounted for system aberrations (Note S5), with the same number of iterations for both patch and RL deconvolution, equal to the number of views. Figs. 3, *e* and *f* show the 3D reconstructions after 19 and 37 iterations, accurately reflecting bead size and shape. The 2D xy-slices at $z = 0$ exhibit similar line profiles and identical full-width half-maximum for both methods. A library of bead reconstructions is presented in Note S4.1.

T-cell data were acquired using highly inclined and laminated optical sheet illumination (HiLO) in both MLA configurations. Again, the number of iterations matched the number of views. The final RL deconvolution result was used as the reference for mean squared error (MSE) calculation. Convergence based on MSE are shown in Figs. 3, *b* and *c*, indicating that patch deconvolution reached results comparable to RL deconvolution in high signal conditions (19 MLA: 8.4×10^{-4}; 37 MLA: 1.8×10^{-3}). Pixel-wise convergence across depth is shown in Fig. 3, *g* and *h*, demonstrating that patch deconvolution accurately reconstructed cell morphology across the field of view within the axial range of a whole cell, albeit with slightly elevated background noise. Runtime benchmarking was performed on an Nvidia RTX 4060 GPU and an Intel i7-13700K CPU (Fig. 3 *d*), using the same number of iterations for both setups. With 37 iterations, patch deconvolution achieved runtimes of 7 ms for the 19 MLA setup and 8 ms for the 37 MLA setup. Reducing the iteration to 19 further halves the reconstruction time to approximately 4 ms in 19 MLA setup, equivalent to 250 reconstructions per second. A library of cell reconstructions is presented in Note S4.2.

Size and order of patches

Next, we evaluated two key parameters in patch deconvolution using simulated data with known ground truth: (1) the order in which the patches are processed and (2) the

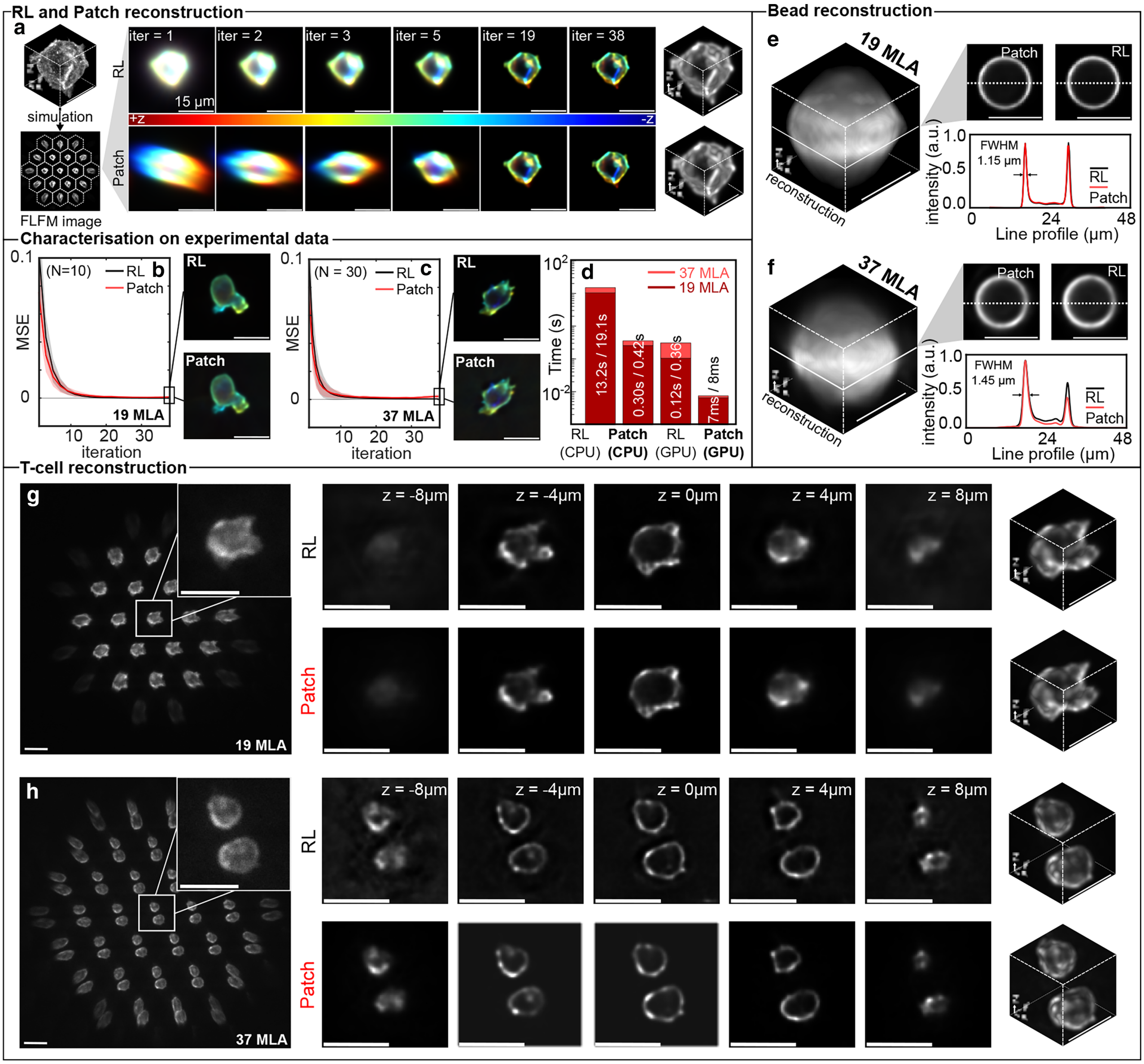

FIGURE 3 Validation on static data. (*a*) Comparison of RL (*top row*) and patch (*bottom row*) deconvolution on the same simulated FLFM image with 19 subaperture views. (*b* and *c*) MSE between RL and patch deconvolution for experimental T-cell data using 19 and 37 MLA configurations; RL results at the 19th and 37th iterations were used as reference. (*d*) Runtime comparison of both methods over 37 iterations on CPU and GPU for 19 and 37 MLA cases. (*e* and *f*) Bead reconstruction comparison (volume, 2D slice, and line profile) between RL and patch deconvolution for a 15-μm bead. (*g* and *h*) T-cell reconstructions with 19 and 37 MLAs show consistent results between methods across the full cell depth. All scale bars represent 15 μm. The shaded region represents ± 1 standard deviation.

size of the patch, to assess their impact on reconstruction quality. A square MLA layout was adopted for a simpler patch segmentation. Simulations used 72 confocal-imaged T-cells with Poisson noise added to achieve varying PSNR levels from 1dB to 80 dB in the FLFM images (Fig. 4 *a*).

Four patch processing orders were tested (Fig. 4 *b*): “out-in” (spiraling from the outermost views inward), “in-out” (the reverse), “grid” (raster scan), and “random.” Each patch was used once, so the number of iterations equaled the number of patches p (Fig. 4 *c*). Fig. 4 *e* shows that “random” converging slightly faster than the others, but consideration of the entire PSF shows no difference. This is also shown in similar reconstruction quality across PSNR levels from 1 dB to 80 dB in Fig. 4 *e*. Fig. 4 *f* shows that the reconstructed PSNR was always higher compared with the input FLFM image due to multiview fusion through deconvolution, but independent of patch order. Therefore, if an early stopping is needed for a faster reconstruction, the “random” order can be used.

The size of patch is another important factor. Up until this point, we have considered patch sizes containing

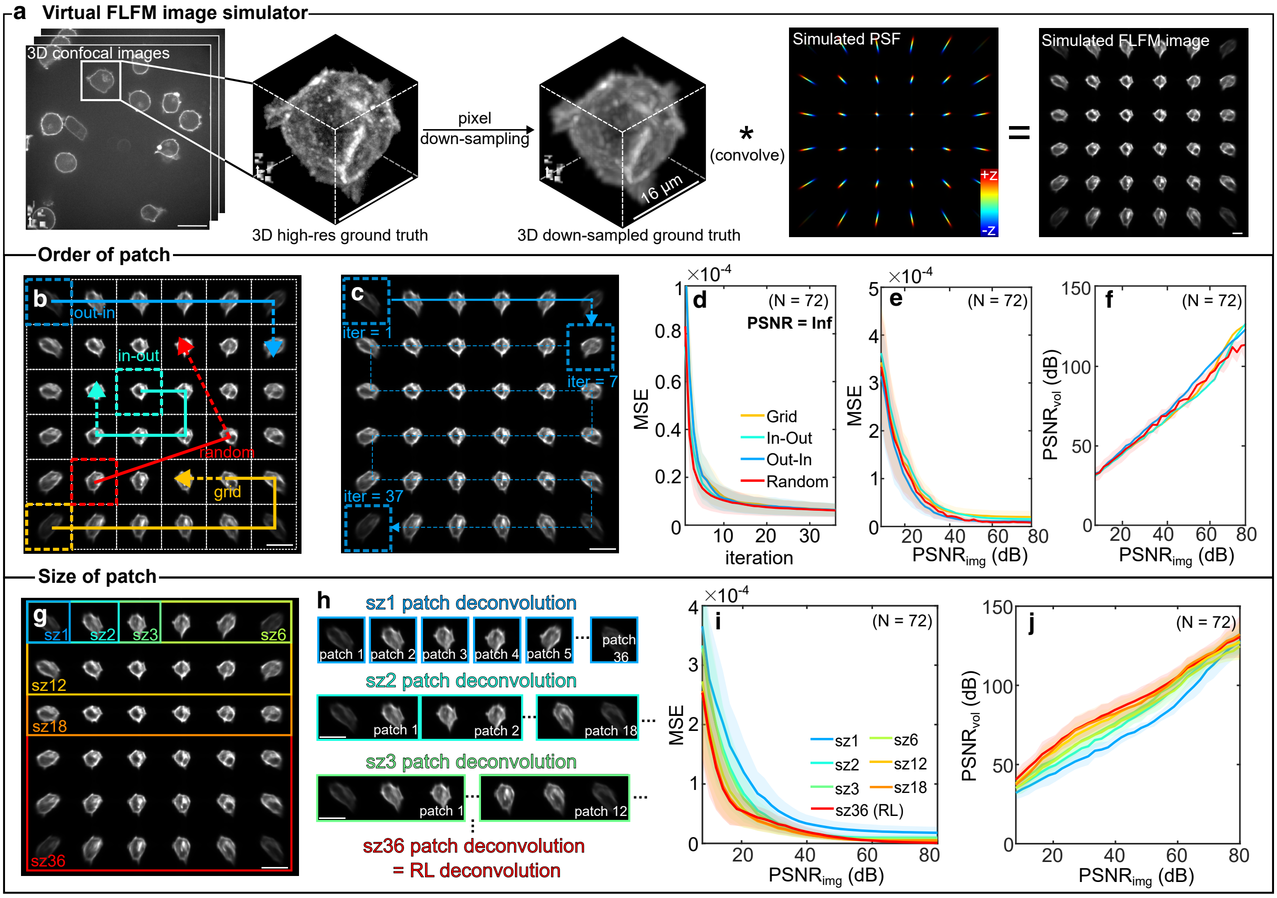

FIGURE 4 Size and order of patches. (*a*) Workflow of the virtual FLFM simulator. High-resolution 3D confocal images are downsampled and convolved with simulated FLFM PSFs to generate test data. (*b*) Different order of patch deconvolution: *grid*, *in-out*, *out-in*, and *random* orders. (*c*) Visualization of the *out-in* deconvolution process. (*d*) Convergence comparison using different patch orders under a noise-free condition. (*e* and *i*) MSE between ground truth and patch deconvolution results at final iteration across different peak signal/noise ratios (PSNRs). (*f* and *j*) PSNR of reconstructed volumes at final iteration versus PSNR of the simulated FLFM image. (*g*) Deconvolution using various patch sizes (*sz1* to *sz36*); *sz36* represents full-image (RL) deconvolution. (*h*) Visualization of patch deconvolution across patch sizes. All scale bars represent 15 μm. The shaded region represents ± 1 standard deviation.

only one FLFM perspective view (*sz1* in Fig. 4 *g*). However, patches may include multiple perspectives, such as *sz2* (2 views), *sz3* (3 views), up to *sz36*, representing full-image RL deconvolution (Fig. 4 *h*). We tested different patch sizes over 36 iterations (equal to the number of views) using a "random" order. MSE comparisons across PSNRs (Fig. 4 *i*) revealed that *sz36* was most robust to noise, whereas *sz1* was least. Therefore, for quantitative analyses in noisy environments, larger patch sizes are preferred. The largest improvement occurred between *sz1* and *sz2*. This trend is also shown in PSNR results (Fig. 4 *j*). Thus, *sz2* offers a practical trade-off between speed and reconstruction quality.

Validation on flow cell data

Next, we evaluated the performance of patch deconvolution under flow conditions. Membrane-labeled T-cells were imaged at 300 events/s (0.5-ms exposure, 16-bit mode, 1000 × 1000 image) (Fig. 5 *a*) and 1500 events/s (0.1-ms exposure, 8-bit mode, 1000 × 1000 image). In the experimental setup, several optical aberrations, including defocus from sample drift within the microfluidic channel and spherical aberration caused by refractive index mismatches, can cause discrepancies between simulated and experimental PSFs. To mitigate this, a hybrid PSF approach (Note S5, Fig. S12) was used, which incorporated experimental information into the simulated model by adjusting the spatial positioning of the PSF based on experimental data. Because computing a hybrid PSF for every image is not practical for large experimental data sets, a PSF library was simulated in advance, and each frame was assigned a specific PSF. At 0.5-ms exposure, the FLFM image PSNR ranged from 18 to 22 dB, slightly lower than typical short-exposure microscopy (46) due to signal division across views. Despite this, patch deconvolution closely matched RL deconvolution in reconstruction accuracy (Fig. 5 *c*). Even at 0.1 ms, 3D reconstructions remained largely consistent, though patch deconvolution exhibited higher background noise (Note S4.3). Besides the morphology comparison,

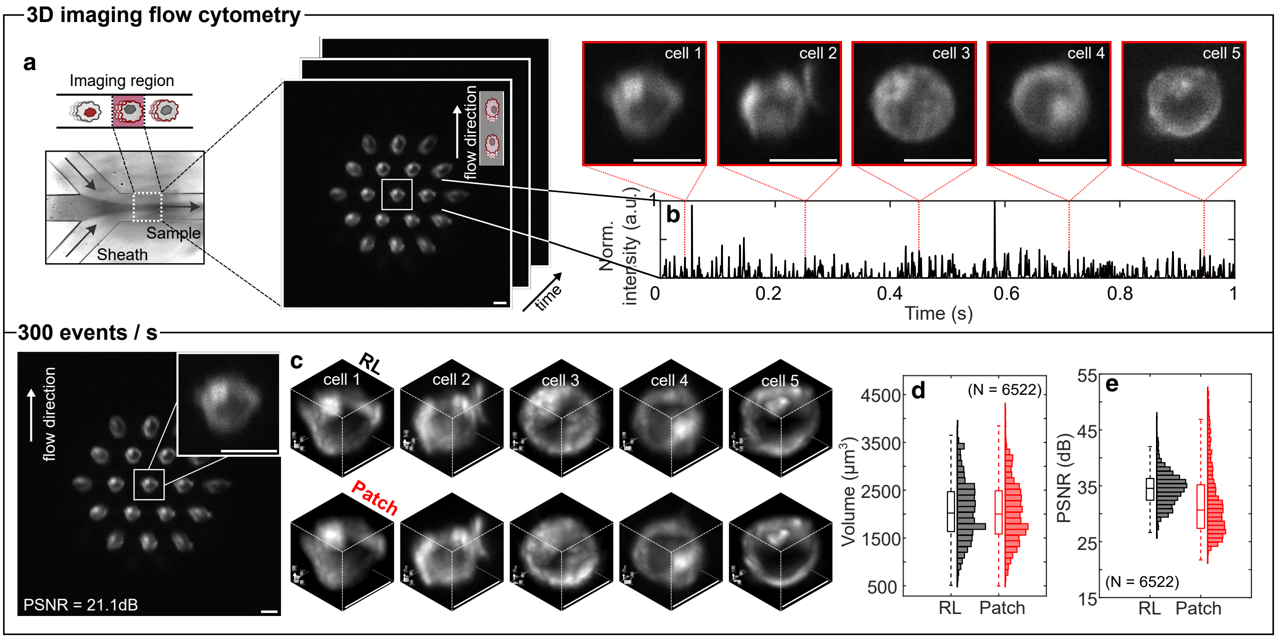

FIGURE 5 Validation on flow cell data. (*a*) Schematic of 3D light-field flow cytometry. (*b*) Sum of normalized intensity in the central view over 1 s at 0.5-ms exposure, highlighting five example cells. (*c*) 3D volume renderings of five cells reconstructed using patch and RL deconvolution at 0.5-ms exposure. (*d*) Comparison of cell volumes from patch and RL deconvolution. (*e*) PSNR comparison of reconstructed volumes between patch and RL deconvolution. All scale bars represent 15 μm. The box plot displays the interquartile range of the data distribution.

reconstructed cell volumes (Fig. 5 *d*), filling the binary masks for reconstructed cells, showed comparable trends between both methods. Combined with PSNR analysis (Fig. 5 *e*), the slightly reduced image quality of patch deconvolution did not compromise the accuracy of quantitative analysis, thereby enabling sorting performance comparable to RL reconstruction. A library of reconstructed cells in flow is shown in Note S4.3.

DISCUSSION

Although patch deconvolution shows great promise for high-throughout 3D imaging cytometry, there are three key limitations should be considered: 1) physical limitations on reconstruction time, 2) limited FOV size for having spatial isolation between patches, and 3) being less robust to high noise conditions. Although faster GPUs can also bring RL reconstruction into the order of milliseconds, patch deconvolution always provides an intrinsic and consistent *p*-fold speedup. Practically, however, reconstruction times are also limited by data transfer between RAM and GPU, code efficiency, and overheads from instruction calls. In addition to the implemented-level limitations, patch deconvolution faces challenges in handling overlapping views, where it becomes difficult or impossible to segment distinct patches. This issue can be avoided by placing an iris at the image plane to restrict the FOV, thus eliminating overlap. Moreover, patch deconvolution is less robust to noise, indicated by images under 0.1 ms exposure and 8-bit mode (Note S4.3), as each update is based on noisier, less consistent statistics. Improved labeling strategies or low-noise cameras are therefore essential for a higher acquisition speed.

We also note that deep learning-based methods indicate that patch-based processing is a promising direction. Recent DL reconstruction frameworks have adopted image patches rather than full views for training, achieving a faster training and a better convergence (20,30). This trend suggests that, regardless of whether one employs DL-based or physics-based strategies, patch-level processing is likely to play a key role in further advancing RL deconvolution in experimental data reconstruction.

CONCLUSION

Here, we have introduced and validated patch deconvolution as a computationally efficient algorithm for 3D cell reconstruction in FLFM at a rate of 100–200 reconstructions per second. Inspired by OSEM (40) and efficient Bayesian multiview deconvolution (39), patch deconvolution speeds up reconstruction by a factor of approximately p, where p denotes the number of views in the FLFM image. A comparative summary of performance across algorithms discussed in the introduction is provided in Table 1. This throughput enables millions of cells to be imaged and timely reconstructed in a single experiment, overcoming a major barrier to the widespread adoption of 3D IFC in life science.

Patch deconvolution enables millisecond-scale 3D reconstructions, achieving cell sorting speeds comparable to the

TABLE 2 Comparison of Different Imaging Flow Cytometry Methods

Instrument	3D	Resolution	Range	Acquisition Speed	Reconstruction Speed	Simplicity	Ref.
This work (19/37MLA)	✓	1.3 μm	whole cell	1500	100–200	++++	–
FLFM (3MLA)	✓	600 nm	subcellular	≤5000	5–10	++++	Hua et al. (20)
Radiofrequency-tagged emission	×	1.55 μm	whole cell	15,000	15,000	+++	Schraivogel et al. (7)
Light-sheet microscopy	✓	1.8 μm	whole cell	10–20	10–20	+++	Kumar et al. (11)
Widefield illumination with PSF engineering	✓	300 nm	single molecule	250	250	++++	Weiss et al. (15)
Lattice light-sheet microscopy	✓	1 μm	whole cell	<10	<10	++	Fan et al. (10)
Scanning light-sheet illumination	✓	2 μm	whole cell	500	500	++	Han et al. (12)
Light-sheet microscopy on chip	✓	300 nm	whole cell	<10	<10	++	Sala et al. (13)
Commercial system (ImageStream)	×	500 nm	whole cell	2000	2000	++++	George et al. (47)

Comparison of different imaging flow cytometry methods based on their capability for 3D imaging, resolution, detection range, acquisition speed (events per second), reconstruction speed (events per second), and simplicity of setup. The reconstruction speed here is proportional to sorting speed. The system resolution and imaging range are discussed in Note S1.2, and the acquisition speed is discussed in Fig. S10.

fastest reported methods (Table 2) while relying on a simpler microscope setup and reconstruction algorithm. This significantly increases the competitiveness of LFC.

Across both static and flow data sets and under varied noise conditions, patch deconvolution produced reconstructions that were comparable to RL in resolution, morphology, and cell volumes, as shown in Video S1, thus having negligible impact on sorting accuracy. Since the only difference lies in matrix size, patch deconvolution requires no changes to existing hardware or software systems and remains fully compatible with other optimization techniques. Patch deconvolution is thus a practical and powerful addition to the 3D LFC. With the acceleration ability, it opens new opportunities for cell sorting based on complex spatial features (2), such as protein co-localization (8) or rare subcellular patterns (Note S6), within large populations. These capabilities are vital for applications in flow cytometry where both precision and throughput are critical.

DATA AND CODE AVAILABILITY

Codes and data in support of this study can be found in the following locations:

- FLFM simulation and patch deconvolution code: https://github.com/binfu0728/patch_deconvolution.
- Raw data and code for all figures: https://doi.org/10.5281/zenodo.15649030.

ACKNOWLEDGMENTS

The authors would like to thank Kevin O'Holleran for valuable discussions.

Funding was from the Center for Doctoral Training in Connected Electronic and Photonic Systems (CEPS) (EP/S022139/1) and CAMS Innovation Fund for Medical Sciences (CIFMS) (2018-I2M-2-002).

AUTHOR CONTRIBUTIONS

B.F. and S.F.L. conceived the project. D.K., T.P.J.K., L.E.W., R.A.F., and S.F.L. oversaw the project. C.L.J., D.H., C.O.'B.-B., and V.J. prepared and stained the cell samples. B.F. and C.L.J. took experimental data. B.F., C.L.J., and S.Y. built a Fourier light-field microscope. C.L.J and H.Y. built the flow cytometer. B.F. performed simulations, reconstructions, and analysis. B.F. and S.F.L. wrote the manuscript with input from all authors.

DECLARATION OF INTERESTS

S.F.L. is a co-founder and shareholder in ZOMP, a biomedical devices company developing spatial flow cytometry.

SUPPORTING MATERIAL

Supporting Material can be found online at https://doi.org/10.1016/j.bpj.2026.01.034.

REFERENCES

1. Cossarizza, A., H. D. Chang, …, A. Zychlinsky. 2019. Guidelines for the use of flow cytometry and cell sorting in immunological studies (second edition). *Eur. J. Immunol.* 49:1457–1973.
2. Han, Y., Y. Gu, …, Y. H. Lo. 2016. Review: Imaging technologies for flow cytometry. *Lab Chip.* 16:4639–4647.
3. Rees, P., H. D. Summers, …, M. Doan. 2022. Imaging flow cytometry. *Nat. Rev. Methods Primers.* 2:86.
4. Stavrakis, S., G. Holzner, …, A. deMello. 2019. High-throughput microfluidic imaging flow cytometry. *Curr. Opin. Biotechnol.* 55:36–43.
5. Mastoridis, S., G. M. Bertolino, …, M. Martinez-Llordella. 2018. Multiparametric analysis of circulating exosomes and other small extracellular vesicles by advanced imaging flow cytometry. *Front. Immunol.* 9:1583.
6. Rane, A. S., J. Rutkauskaite, …, S. Stavrakis. 2017. High-Throughput Multi-parametric Imaging Flow Cytometry. *Chem.* 3:588–602.
7. Schraivogel, D., T. M. Kuhn, …, L. M. Steinmetz. 2022. High-speed fluorescence image-enabled cell sorting. *Science.* 375:315–320. https://www.science.org.
8. Ram, S., D. Kim, …, E. S. Ward. 2012. 3D Single Molecule Tracking with Multifocal Plane Microscopy Reveals Rapid Intercellular Transferrin Transport at Epithelial Cell Barriers. *Biophys. J.* 103:1594–1603.
9. Woodcroft, B. J., L. Hammond, …, N. A. Hamilton. 2009. Automated organelle-based colocalization in whole-cell imaging. *Cytometry. A.* 75:941–950.
10. Fan, Y. J., H. Y. Hsieh, …, B. C. Chen. 2021. Microfluidic channel integrated with a lattice lightsheet microscopic system for continuous cell imaging. *Lab Chip.* 21:344–354.

11. Kumar, P., P. Joshi, …, P. P. Mondal. 2022. Light sheet based volume flow cytometry (VFC) for rapid volume reconstruction and parameter estimation on the go. *Sci. Rep.* 12:78.

12. Han, Y., R. Tang, …, Y.-H. Lo. 2019. Cameraless high-throughput three-dimensional imaging flow cytometry. *Optica.* 6:1297.

13. Sala, F., M. Castriotta, …, A. Bassi. 2020. High-throughput 3D imaging of single cells with light-sheet fluorescence microscopy on chip. *Biomed. Opt. Express.* 11:4397–4407.

14. Lai, Q. T. K., G. G. K. Yip, …, K. K. Tsia. 2021. High-speed laser-scanning biological microscopy using FACED. *Nat. Protoc.* 16:4227–4264.

15. Weiss, L. E., Y. Shalev Ezra, …, Y. Shechtman. 2020. Three-dimensional localization microscopy in live flowing cells. *Nat. Nanotechnol.* 15:500–506.

16. Guo, C., W. Liu, …, S. Jia. 2019. Fourier light-field microscopy. *Opt. Express.* 27:25573–25594.

17. Sims, R. R., S. Abdul Rehman, …, K. O'Holleran. 2020. Single molecule light field microscopy. *Optica.* 7:1065.

18. Scrofani, G., J. Sola-Pikabea, …, M. Martínez-Corral. 2018. FIMic: design for ultimate 3D-integral microscopy of in-vivo biological samples. *Biomed. Opt. Express.* 9:335–346.

19. Galdón, L., G. Saavedra, …, E. Sánchez-Ortiga. 2022. Fourier light-field microscopy: a practical design guide. *Appl. Opt.* 61:2558–2564.

20. Hua, X., K. Han, …, S. Jia. 2024. Light-field flow cytometry for high-resolution, volumetric and multiparametric 3D single-cell analysis. *Nat. Commun.* 15:1975.

21. Lucy, L. B. 1974. An iterative technique for the rectification of observed distributions. *Astron. J.* 79:745–754.

22. Richardson, W. H. 1972. Bayesian-Based Iterative Method of Image Restoration. *J. Opt. Soc. Am.* 62:55–59.

23. Shepp, L. A., and Y. Vardi. 1982. Maximum likelihood reconstruction for emission tomography. *IEEE Trans. Med. Imaging.* 1:113–122.

24. Dempster, A. P., N. M. Laird, and D. B. Rubin. 1977. Maximum Likelihood from Incomplete Data via the EM Algorithm. *J. Roy. Stat. Soc. B.* 39:1–22.

25. Sarder, P., and A. Nehorai. 2006. Deconvolution Methods for 3-D Fluorescence Microscopy Images. *IEEE Signal Process. Mag.* 23:32–45.

26. Biggs, D. S., and M. Andrews. 1997. Acceleration of iterative image restoration algorithms. *Appl. Opt.* 36:1766–1775.

27. Holmes, T. J., and Y.-H. Liu. 1991. Acceleration of maximum-likelihood image restoration for fluorescence microscopy and other noncoherent imagery. *J. Opt. Soc. Am. A.* 8:893–907.

28. Guo, M., Y. Li, …, H. Shroff. 2020. Rapid image deconvolution and multiview fusion for optical microscopy. *Nat. Biotechnol.* 38:1337–1346.

29. Wu, S., W. Liu, …, S. Jia. 2025. PEARL: projection-estimation accelerated Richardson-Lucy deconvolution for rapid volumetric imaging. *Opt. Express.* 33:22685–22698.

30. Yi, C., L. Zhu, …, P. Fei. 2023. Video-rate 3D imaging of living cells using Fourier view-channel-depth light field microscopy. *Commun. Biol.* 6:1259.

31. Wang, Z., L. Zhu, …, P. Fei. 2021. Real-time volumetric reconstruction of biological dynamics with light-field microscopy and deep learning. *Nat. Methods.* 18:551–556.

32. Wagner, N., F. Beuttenmueller, …, A. Kreshuk. 2021. Deep learning-enhanced light-field imaging with continuous validation. *Nat. Methods.* 18:557–563.

33. Lu, Z., M. Jin, …, Q. Dai. 2025. Physics-driven self-supervised learning for fast high-resolution robust 3D reconstruction of light-field microscopy. *Nat. Methods.* 22:1545–1555.

34. Su, C., Y. Gao, …, B. Xiong. 2023. AutoDeconJ: A GPU-accelerated ImageJ plugin for 3D light field deconvolution with optimal iteration numbers predicting. *Bioinformatics.* 39:btac760.

35. Bruce, M. A., and M. J. Butte. 2013. Real-time GPU-based 3D Deconvolution. *Opt. Express.* 21:4766–4773.

36. Zanella, R., G. Zanghirati, …, G. Vicidomini. 2013. Towards real-time image deconvolution: Application to confocal and STED microscopy. *Sci. Rep.* 3:2523.

37. Schmid, B., and J. Huisken. 2015. Real-time multi-view deconvolution. *Bioinformatics.* 31:3398–3400.

38. Ingaramo, M., A. G. York, …, G. H. Patterson. 2014. Richardson–Lucy Deconvolution as a General Tool for Combining Images with Complementary Strengths. *ChemPhysChem.* 15:794–800.

39. Preibisch, S., F. Amat, …, P. Tomancak. 2014. Efficient Bayesian-based multiview deconvolution. *Nat. Methods.* 11:645–648.

40. Hudson, H. M., and R. S. Larkin. 1994. Accelerated Image Reconstruction Using Ordered Subsets of Projection Data. *IEEE Trans. Med. Imaging.* 13:601–609.

41. Kempen, G. M. V., and L. J. V. Vliet. 2000. The influence of the regularization parameter and the first estimate on the performance of Tikhonov regularized non-linear image restoration algorithms. *J. Microsc.* 198:63–75.

42. Dey, N., L. Blanc-Feraud, …, J. Zerubia. 2006. Richardson-Lucy algorithm with total variation regularization for 3D confocal microscope deconvolution. *Microsc. Res. Tech.* 69:260–266.

43. Stefanoiu, A., J. Page Vizcaíno, and T. Lasser. 2019. oLaF: A flexible 3D reconstruction framework for light field microscopy. *arXiv.* https://arxiv.org/abs/1910.08419.

44. Fu, B., E. E. Brock, …, S. F. Lee. 2024. RASP: Optimal Single Puncta Detection in Complex Cellular Backgrounds. *J. Phys. Chem. B.* 128:3585–3597.

45. Challa, P. K., T. Kartanas, …, T. P. J. Knowles. 2017. Microfluidic devices fabricated using fast wafer-scale LED-lithography patterning. *Biomicrofluidics.* 11:014113.

46. Chen, F., J. Liu, …, H. Liao. 2020. An accurate and universal approach for short-exposure-time microscopy image enhancement. *Comput. Med. Imaging Graph.* 83:101743.

47. George, T. C., D. A. Basiji, …, P. J. Morrissey. 2004. Distinguishing modes of cell death using the ImageStream® multispectral imaging flow cytometer. *Cytometry. A.* 59:237–245. https://doi.org/10.1002/cyto.a.20048.

Biophysical Journal, Volume *125*

Supplemental information

Patch deconvolution for Fourier light-field microscopy

Bin Fu, Caroline L. Jones, Daniel Heraghty, Shengbo Yang, Caitlin O'Brien-Ball, Victoria Junghans, Haowei Yang, David Klenerman, Tuomas P.J. Knowles, Lucien E. Weiss, Ricardo A. Fernandes, and Steven F. Lee

Supplementarty Information: Patch deconvolution for Fourier light-field microscopy

Bin Fu[1], Caroline L. Jones[1], Daniel Heraghty[1], Shengbo Yang[1], Caitlin O'Brien-Ball[2], Victoria Junghans[2], Haowei Yang[1], David Klenerman[1], Tuomas P.J. Knowles[1], Lucien E. Weiss[3], Ricardo A. Fernandes[2], and Steven F. Lee[1,*]

[1]Yusuf Hamied Department of Chemistry, University of Cambridge, Lensfield Road, Cambridge, CB2 1EW, UK
[2]Chinese Academy of Medical Sciences (CAMS) Oxford Institute (COI), University of Oxford, Oxford, OX3 7BN, UK
[3]Department of Engineering Physics, Polytechnique Montréal, Montréal, Québec, H3T 1J4, Canada
[*]Correspondence: sl591@cam.ac.uk

S1 OPTICAL SYSTEM DESIGN

S1.1 System schematic

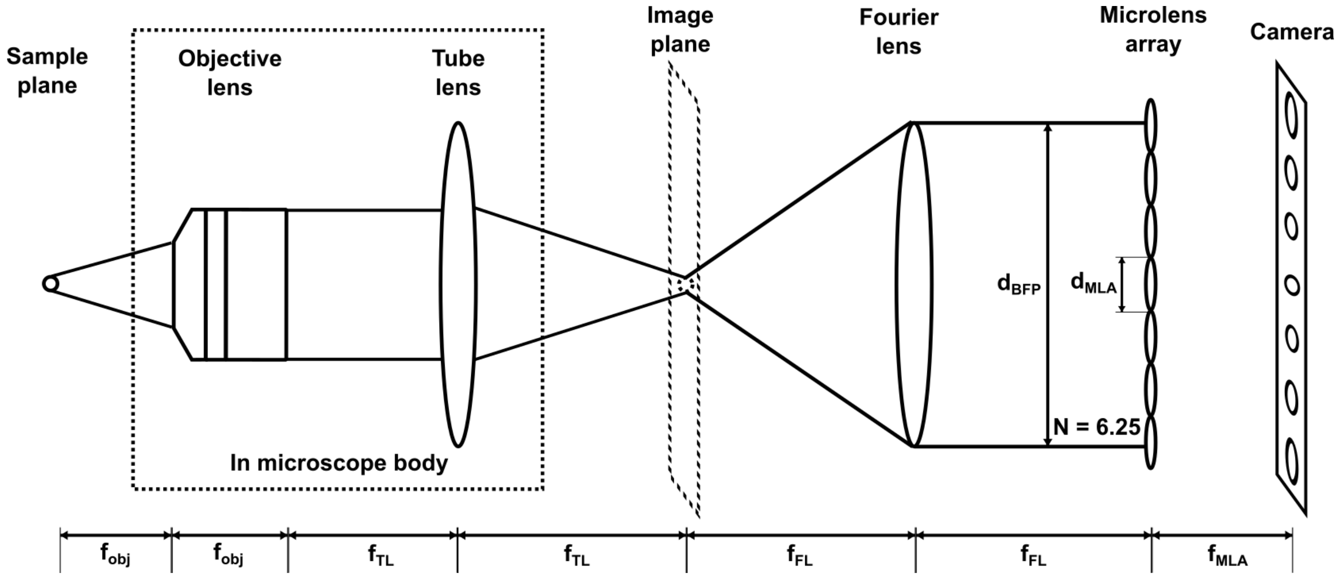

Figure S1: **Schematic layout of FLFM.** The simplified system of a FLFM microscope with objective lens, tube lens, Fourier lens, MLA and the camera. Multiple perspectives of the sample are formed on the camera due to the MLA. N = 6.25 is the setup for 37 MLA included in the back focal plane (BFP)

The lateral resolution, defined in Eqn. S1, and the axial resolution, defined in Eqn. S2 (1), are two key parameters in the FLFM system design, where N is the ratio between the diameter of back focal plane (BFP) (d_{BFP}) and the diameter of a microlens (d_{MLA}) and NA represents the objective lens's numerical aperture. Since the diameter of BFP is divided by N microlenses, the effective numerical aperture ($\mathrm{NA}_{\mathrm{MLA}}$) for each perspective image in FLFM can be described as NA/N. Therefore, the lateral resolution can be represented in S1.

$$R_{\mathrm{xy}} = \frac{\lambda}{2\mathrm{NA}_{\mathrm{MLA}}} = \frac{\lambda N}{2\mathrm{NA}} \tag{S1}$$

The equation for axial resolution, derived from ray optics, has been adapted for a hexagonal microlens array (MLA) pattern. In the case of a 37 MLA configuration (Fig. S1), the transformation from axial displacement in the sample plane to lateral displacement due to MLA on the camera is represented by $\tan\theta$ (1). Here, θ is the angle formed between f_{FL} and half the

radius of the BFP after the Fourier lens. This angle represents the average axial shift across all perspectives on camera. Hence, $\tan\theta = \frac{N}{4} \times \frac{d_{\mathrm{MLA}}}{f_{\mathrm{FL}}}$. The axial resolution can be determined by back-projecting the lateral resolution to its corresponding axial distance in the sample plane, *i.e.*the minimum axial separation at which two emitters can be resolved. This minimum distance can be calculated using Eqn. S2 using $d_{\mathrm{MLA}} = 2f_{\mathrm{MLA}}\mathrm{NA}_{\mathrm{MLA}}$

$$R_z = \frac{\lambda}{2\mathrm{NA}_{\mathrm{MLA}}} \times \frac{f_{\mathrm{FL}}}{f_{\mathrm{MLA}}} \times \frac{1}{\tan\theta} \times \left(\frac{f_{obj}}{f_{TL}}\right)^2 = \frac{\lambda N}{\mathrm{NA}^2} \tag{S2}$$

The depth-of-field (DoF) defined in Eqn. S3 is also an important factor in the system design. It is described by the sum of the wave and geometrical optical depths of fields where δ is the pixel size of the camera and M_{T} is the total magnification of the system (2).

$$DoF = 2\lambda\frac{N^2}{\mathrm{NA}^2} + \frac{\delta}{M_{\mathrm{T}}}\frac{N}{\mathrm{NA}} \tag{S3}$$

The field-of-view (FoV) is another crucial consideration in system design to ensure complete cell capture, which is determined by M_{T} and the pitch of MLA.

$$\mathrm{FoV} = \frac{d_{\mathrm{MLA}}}{M_{\mathrm{T}}} \tag{S4}$$

To image an entire cell in flow, several design requirements must be met. First, lateral needs to be within 1-2 µm while axial resolution needs to be within 2-3 µm to achieve similar lateral and axial resolution sufficient for capturing cell morphology. This requirement sets an upper limit on N in BFP. Second, the DoF must exceed 15 µm to image the whole cell while allowing for some movement within the microfluidic channel, establishing a lower limit on N in BFP. Based on these constraints, we validated the patch deconvolution using 19 MLA and 37 MLA setup.

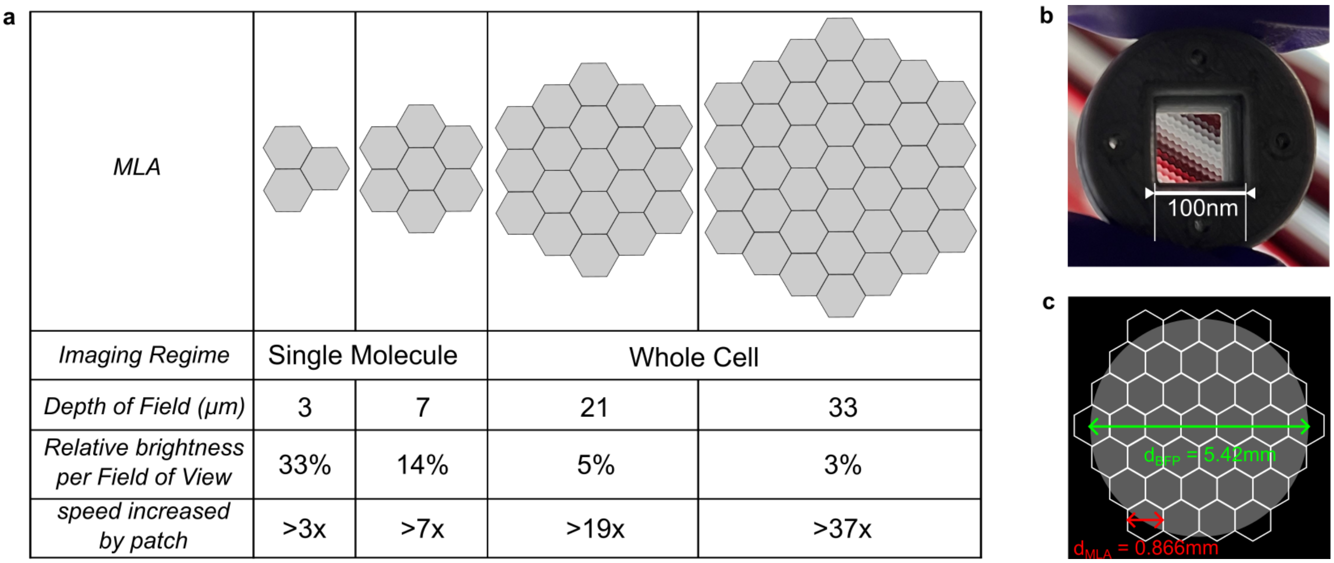

Figure S2: **Parameter comparison. a** Diagram illustrating different configurations of MLA used for various imaging regimes, including Single Molecule and Whole Cell imaging. By varying in size and number, the depth of field, relative brightness per field of view, and speed increase achieved by patching are different for different configurations. **b** Photograph of MLA in SM1 compatible retaining ring. **c** Depiction of BFP size and the number of MLA contained within. Fourier lens of 125 mm produces BFP diameter of 5.42 mm covering 6.25 lenses for 37 MLA setup.

S1.2 System design parameters

The specific design parameters for 19 MLA and 37 MLA setup is listed in the Table S1. The only change between two setups is the choice of Fourier lens: 100 mm for a 19 MLA setup and 125 mm for a 37 MLA setup, which leads to a different BFP size (4.33 mm for 19 MLA setup and 5.42 mm for 37 MLA setup).

Variable	Symbol	Value (mm)
Numerical aperture	NA	1.3
Objective magnification	M_{obj}	60
Tube lens focal length (mm)	f_{TL}	180
Objective focal length (mm)	$f_{\text{obj}} = \frac{f_{\text{TL}}}{M_{\text{obj}}}$	3.0
Fourier lens focal length (mm)	f_{FL}	100/125
MLA focal length (mm)	f_{MLA}	36.7
MLA height (mm)	d_{MLA}	0.866
Pixel size (µm)	δ	6.5
Emission wavelength (nm)	λ	680
Total magnification	$M_{\text{T}} = \frac{f_{\text{MLA}} f_{\text{TL}}}{f_{\text{obj}} f_{\text{FL}}}$	22/17.6
Diameter BFP (mm)	$d_{\text{BFP}} = \frac{2\text{NA} f_{\text{obj}} f_{\text{FL}}}{f_{\text{TL}}}$	4.33/5.42
Number of MLA in BFP	$N = \frac{d_{\text{BFP}}}{d_{\text{MLA}}}$	5/6.25

Table S1: **Summary of design parameters in the FLFM optical system design.** The first column shows the parameter from 19 MLA setup while the second column shows the parameters from 37 MLA setup.

With such design parameters and using the equations discussed in the previous section, the performance parameter can be concluded in the Table S2 where the first column corresponds to 19 MLA setup and the second corresponds to the 37 MLA setup

Variable	Symbol	Value (mm)
Lateral resolution (µm)	R_{xy}	1.3/1.6
Axial resolution (µm)	R_{z}	2.0/2.6
Depth of field (µm)	DoF	21.2/33.2
Field Of View (µm)	FoV	39.4/49.2

Table S2: **Summary of performance of the system based on the design parameters.** The first column shows the parameter from 19 MLA setup while the second column shows the parameters from 37 MLA setup.

S2 IMAGE FORMATION AND RECONSTRUCTION MODEL

S2.1 Forward imaging model

The forward projection model is essential for simulating FLFM images. In this paper, the forward projection model is derived from the approach presented in (3).

Initially, the phase $\Phi(u, v)$ at the BFP, resulting from displacements of a point source from the focal point, is given by Eqn. S5. (x,y,z) denotes the coordinate at the object plane, and (u, v) denotes the coordinate at the BFP. n_s is the refractive index of the sample. k is the wavevector defined as $k = \frac{2\pi}{\lambda_0}$, and f is the focal length of the objective lens. Here, (x, y) in the BFP plane is mapped directly to (u, v) in the camera plane, since the ray height is preserved during the mapping from the object plane to the BFP plane (4).

$$\Phi(u, v; x, y, z) = \frac{n_s k}{f}\left[(xu + yv) + z\sqrt{f^2 - u^2 - v^2}\right] \tag{S5}$$

In the normalized coordinate system at the BFP, the normalized radius, ρ, is defined such that $\rho^2 = u^2 + v^2 = 1$. According to the small angle approximation, the normalized radius of the BFP is approximately equal to $f \cdot \text{NA}$. When accounting for the mismatch between the sample's refractive index and the objective lens, the focal length can be derived as $f = n_s/\text{NA}$. Therefore, Eqn. S5 becomes:

$$\Phi(u, v; x, y, z) = k\text{NA}\,(xu + yv) + n_s zk\sqrt{1 - \left(\frac{\text{NA}\rho}{n_s}\right)^2} \tag{S6}$$

By introducing a microlens array at the BFP, which acts as a phase modulator, the phase $\Phi(u, v)$ in Eqn. S6 is modified to Eqn. S7. In this equation, $\Phi_{mla}(u, v)$ represents the extra phase from the microlens array.

$$\Phi'(u, v; x, y, z) = \Phi(u, v; x, y, z) + \Phi_{mla}(u, v) \tag{S7}$$

The electric field $E(x, y)$ at the camera can be modeled using Fresnel propagation over the distance f_{mla} from the BFP. The square of the magnitude of the electric field $E(x, y)$, assuming circularly polarised, is the point spread function (PSF) $h(x, y)$ of the system, described in Eqn. S8. Here, (u, v) in the BFP plane is mapped directly to (x, y) in the camera plane (4)

$$h(u, v; x, y, z) = \left\{\mathcal{F}^{-1}\left\{\mathcal{F}\{\exp(\Phi'(u, v; x, y, z))\} \cdot \exp\left(\frac{-j}{4\pi}\lambda f_{mla}(u^2 + v^2)\right)\right\}\right\}^2 \tag{S8}$$

Since FLFM is spatially invariant (5), the PSF $h(u, v; x, y, z)$ can be simplified to $h(u, v; z)$ by ignoring kNA $(xu + yv)$ term in Eqn. S6, where z denotes the location of the point source along the z-axis. Based on this simplified $h(u, v)$, and the corresponding coordinate between sample plane (x, y) and the image plane (u, v), the forward model of FLFM can be represented by Eqn. S9, where $o(x, y; z)$ represents the object distribution function $o(x, y)$ at a specific z-plane and $I(x, y)$ represents the simulated FLFM image.

$$I(x, y) = \int_z h(x, y; z) * o(x, y; z) dz \tag{S9}$$

S2.2 Richardson-Lucy deconvolution for FLFM

To solve this inverse problem (*i.e.*recovering the object from the observed image), we begin with the simplest case where both the observed image $I(x, y)$ and the object $o(x, y)$ are 2D images. Since the noise statistics of an image are dominated by a Poisson process (6), the distribution P of an observed image I at pixel s can be expressed in Eqn. S10. Here, the forward imaging model is represented as $(o * h)(s)$, where s represents the coordinates (x, y).

$$P(I(s)|(o * h)(s)) = \frac{[(o * h)(s)]^{I(s)} e^{-(o*h)(s)}}{I(s)!} \tag{S10}$$

Assuming that the noise is spatially uncorrelated, the likelihood distribution for all pixels in the observed image I, given the object o, can be expressed in Eqn. S11 where S represents the total set of pixel coordinates in the image.

$$P(I|o) = \prod_{s \in S} \left(\frac{[(o * h)(s)]^{I(s)} e^{-(o*h)(s)}}{I(s)!}\right) \tag{S11}$$

To maximize the likelihood distribution in Eqn. S11 with respect to o, we can transform Eqn. S11 to $-\log(P(I|o))$. This transformation converts the maximization problem into a minimization problem, where the objective is to minimize $J_1(o)$, as defined in Eqn. S12.

$$J_1(o) = \sum_s (-I(s) \cdot \log[(o * h)(s)] + (o * h)(s)) \tag{S12}$$

To find the optimal solution for $J_1(o)$, it is equivalent to solve $\frac{\partial J_1(o)}{\partial o} = 0$. By using $g(o)$ representing $(o * h)(s)$, $\frac{\partial J_1(o)}{\partial o} = \frac{\partial J_1(g)}{\partial g} \cdot \frac{\partial g(o)}{\partial o}$. The $\frac{\partial J_1(o)}{\partial o} = 0$ therefore can be expressed in Eqn. S13

$$\frac{I(s)}{g(s)} * h(-s) = \sum_{s \in S} h(s) \tag{S13}$$

Since the PSF h also represents as the probability distribution of a single photon, the right hand side of Eqn. S13 is 1. Therefore, Eqn. S13 can be converted to Eqn. S14

$$\frac{I(s)}{(o * h)(s)} * h(-s) = 1 \tag{S14}$$

To solve Eqn. S14, the iterative maximum-likelihood by expectation maximization (MLEM) algorithm from Richardson (7) and Lucy (8) is given by Eqn. S15 where k represents the iteration index.

$$o_{k+1}(s) = \left\{ \left[\frac{I(s)}{(o_k * h)(s)} \right] * h(-s) \right\} \cdot o_k(s) \tag{S15}$$

The Richardson-Lucy deconvolution (Eqn. S15) in FLFM can be adapted in Eqn. S16

$$o_{k+1}(x, y, z) = \left\{ \left[\frac{I(x, y; z)}{\int_z h(x, y; z) * o(x, y; z) dz} \right] * h(-x, -y; z) \right\} \cdot o_k(x, y, z) \tag{S16}$$

S2.3 Patch deconvolution for FLFM

The major difference between RL deconvolution and patch deconvolution lies in the size of the PSF h, the FLFM image I, and the object o used during the iterative process. In patch deconvolution, the PSF h and the FLFM image I are cropped, resulting in the object also being cropped to match the size of the FLFM or the PSF patch. Consequently, the coordinates in the image, originally denoted by (x, y), are changed to (x_p, y_p), which represent local coordinates in the patch. Thus, Eqn. S16 is modified to Eqn. S17 for patch deconvolution where I_p and h_p represents the pth patch of FLFM image and PSF.

$$o_{k+1}(x_p, y_p, z) = \left\{ \left[\frac{I_p(x_p, y_p; z)}{\int_z o_k(x_p, y_p; z) * h_p(x_p, y_p; z) dz} \right] * h_p(-x_p, -y_p; z) \right\} \cdot o_k(x_p, y_p, z) \tag{S17}$$

In RL deconvolution, a single PSF is used only once, whereas in patch deconvolution, it can be utilized p times, where p is the number of perspectives (*i.e.*patches) in the FLFM image. This repeated use of the same PSF information leads to more frequent updates, resulting in a much faster convergence speed for patch deconvolution compared to RL deconvolution. The patch deconvolution algorithm is fully compatible with the existing RL code. The main adjustment involves changing the input from a single PSF and image to different patches of the image and their corresponding PSFs. The algorithm can be implemented using the existing RL algorithm as follows:

(1). **Patch Extraction**: Crop each view in the image and their corresponding PSFs. The result should be a 4D matrix (m, n, z, p), where (m,n) is the size of each patch, z represents the sampling range along the z-axis, and p is the number of perspectives.

(2). **Initialization**: Initialize the object volume (m, n, z) with all elements set to one.

(3). **Iterative Update**: Call the RL deconvolution function, but use a randomly chosen patch image and the corresponding patch PSF as inputs. Also, set the number of iterations to 1. Then, use another randomly selected patch of image and PSF, along with the updated volume from the previous step, for the next iteration.

(4). **Repeat Iterations**: Repeat step (3) until all patches have been utilized. After completing a pass through all patches, continue iterating by revisiting patches as needed. The iterations can be stopped at any point based on a convergence criteria or after a predetermined number of iterations.

S2.4 Convergence of patch deconvolution

The derivation is based on the work of Broxton (9) and Hudson (10).

As described by Broxton (9), the PSF h in the image formation model without considering noise $I = o * h$ can be discretised and the model can be reformulated as a linear system $y = Hx$, where H is the projection matrix whose elements H_{tj} represent the proportion of light from voxel t in the object volume reaching pixel j in the FLFM image. The FLFM image has the number of pixels $N_x \times N_y$, while the object has the number of voxels $N_x \times N_y \times N_z$. The vectors y and x are the flattened forms of the FLFM image and object volume, respectively.

RL deconvolution is an iterative maximum-likelihood estimation algorithm for finding a feasible solution for x. For the model $y = Hx$, the multiplicative update rule at iteration k can be written as:

$$x_j^{k+1} = x_j^k \sum_{t \in N_p} \frac{y_t}{\mu_t^k} H_{tj}, \quad \text{where} \quad \mu_t^k = \sum_{j \in N_v} H_{tj} x_j^k \tag{S18}$$

In patch deconvolution, only a subset $S_k \subset N_p$ is used at each iteration k, leading to the update:

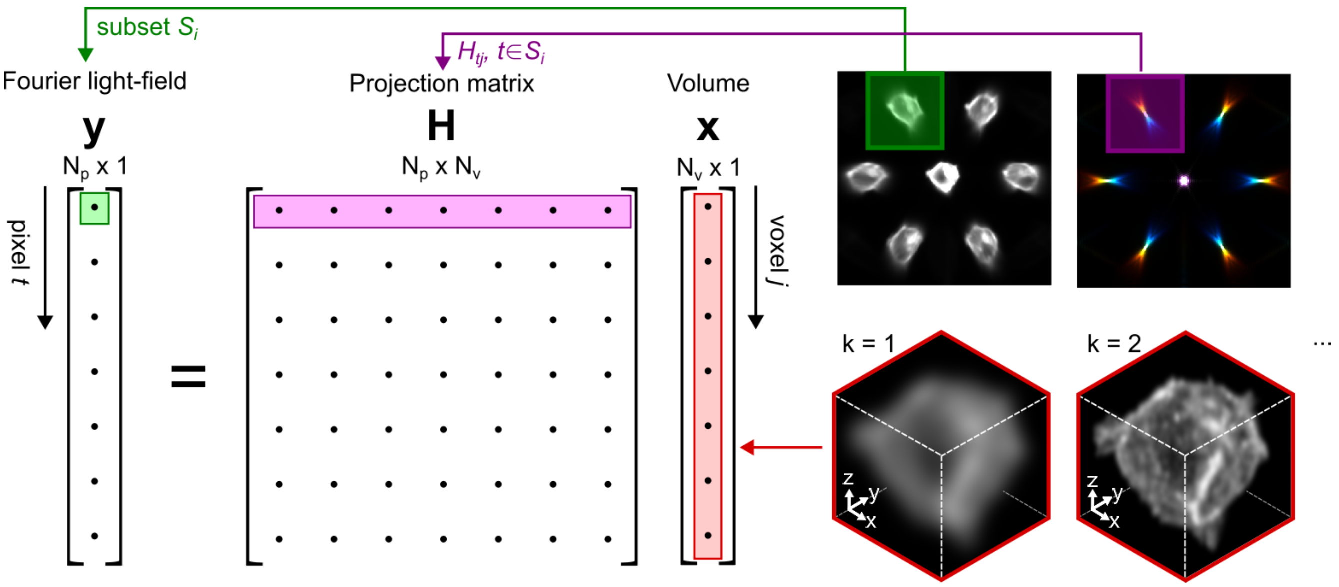

Figure S3: **The discrete patch deconvolution model without noise.** The FLFM image is flattened into a column vector of length $N_p \times 1$, where $N_p = N_x \times N_y$. Each subset S corresponds to a specific group of rows in this vector (*i.e.* $t \in S_i$). Similarly, the object volume is flattened into a vector of length $N_v \times 1$, where $N_v = N_x \times N_y \times N_z$. The projection matrix encodes the proportion of light contributed by each voxel t in the object volume to each pixel j in the FLFM image. During each iteration, a subset (or patch) of the data is used to update the entire volume.

$$x_j^{k+1} = x_j^k \sum_{t \in S_k} \frac{y_t}{\mu_t^k} H_{tj} \tag{S19}$$

To establish convergence of patch deconvolution to the feasible solution, we aim to prove two propositions (10):

1 The Eqn. S19 converges through iterative updates.

2 The Eqn. S19 converges to the global optimum as Eqn. S18, *i.e.*the residual approaches zero at every pixel between forward-projected image and measured image.

For proving two propositions, three assumptions have to be satisfied:

1 All $H_{tj} \geq 0$, and for each t, there exists at least one $H_{tj} > 0$. This ensures each pixel receives contributions from at least one voxel.

2 Since each patch is treated independently and the PSF represents a probability distribution, it holds that $\sum_{t \in S_i} H_{tj} = 1$ for each subset S_i.

3 The ideal solution (*i.e.*ground truth object) is confined within the FoV of the FLFM; that is, the voxel values outside the FoV are zero.

To prove Proposition 1, assume there exists a strictly positive solution $x^* > 0$ such that $y = Hx^*$ and define $0 * \log 0 = 0$ for blank voxels. Define the Kullback–Leibler (KL) divergence at iteration k as:

$$L^k(x; x^*) = -\sum_{j \in N_v} x_j^* \log(\frac{x_j^*}{x_j^k}) \tag{S20}$$

To show the monotonicity of the KL divergence, the change in KL divergence between iterations can be calculated as (10):

$$
\begin{aligned}
\Delta^k &= L^{k+1} - L^k \\
&= \sum_j x_j^* \log(\frac{x_j^{k+1}}{x_j^k}) \\
&= \sum_j x_j^* \log(\sum_{t \in S_k} \frac{y_t}{\mu_t^k} H_{tj}) \\
&\geq \sum_j x_j^* \sum_{t \in S_k} H_{tj} \log(\frac{y_t}{\mu_t^k}) \\
&= \sum_{t \in S_k} \log(\frac{y_t}{\mu_t^k}) \sum_j H_{tj} x_j^* \\
&= \sum_{t \in S_k} y_t \log(\frac{y_t}{\mu_t^k})
\end{aligned}
$$

where the first inequality follows Jensen's inequality and second inequality follows the inequality $\log x \geq 1 - \frac{1}{x}$. Due to the count preservation in Assumption 2, $\sum_{t \in S_k} y_t = \sum_{t \in S_k} \mu_t^k$, Δ^k can be presented as:

$$
\Delta^k = \sum_{t \in S_k} y_t \log(\frac{y_t}{\mu_t^k}) \geq \sum_{t \in S_k} (y_t - \mu_t^k) = 0 \tag{S21}
$$

To show that L^k is bounded above, we use the inequality again together with the count preservation, leading to $L^k(x; x^*) \leq 0$. Thus, L^k is non-decreasing and bounded, completing the proof of Proposition 1.

For Proposition 2, assume that each patch is selected infinitely often. We first demonstrate that the forward-projected image using a single patch PSF converges to the measured data corresponding to that particular perspective view. Specifically, for $t \in S_1$ (*i.e.*each pixel in the first perspective view), we have $\mu_t^{k_1} \to y_t$ as $k_1 \to \infty$, where $k_1 \in K_1 = \{1, n+1, 2n+1, \ldots\}$ denotes the index sequence corresponding to updates involving the first patch, and n is the total number of patches. According to Proposition 1, where Eqn. S21 tends to zero, such convergence occurs if and only if $\mu_t^{k_1} = y_t$ for each $t \in N_p$. This establishes point-wise convergence of the forward-projected image to the measured image for the selected patch. If S_1 now representing the whole FLFM image, this shows the convergence of RL deconvolution.

The next step is to show $\mu_t^{k_1} \to y_t$ for $k_1 \in K_1$, for $t \in N_p$ (*i.e.*each pixel in the entire FLFM image), which can be written as (10):

$$
\begin{aligned}
&|\sum_j H_{tj} x_j^{k_1} - y_t| \\
&\qquad = |\sum_j H_{tj} x_j^{k_i} \prod_{k=k_i}^{k_1-1} (\sum_{t \in S_k} \frac{y_t}{\mu_t^k} H_{tj}) - y_t| \\
&\qquad = |\sum_j H_{tj} x_j^{k_i} \exp\{\sum_{k=k_i}^{k_1-1} \log(\sum_{t \in S_k} \frac{y_t}{\mu_t^k} H_{tj})\} - y_t| \\
&\qquad \leq |\sum_j H_{tj} x_j^{k_i} - y_t| + |\sum_j H_{tj} x_j^{k_i} \{\exp[\sum_{k=k_i}^{k_1-1} \log(\sum_{t \in S_k} \frac{y_t}{\mu_t^k} H_{tj})] - 1\}|
\end{aligned}
$$

The inequality arises from the application of the triangle inequality. Here, $i \in 2, \ldots, n$ and $k_i \in K_i = \{i, n+i, 2n+i, \ldots\}$ is the largest integer less than k_1, which representing the index corresponding to the completion of a full cycle over all patches prior to iteration k_1.

As $k_i \to \infty$, the first term at RHS tends to zero due to convergence of individual patches. The second term also vanishes because the exponential term converges to 1, as a result of pixel-wise convergence $\mu_k^t \to y_t$, which leads to that $\sum_{t \in S_k} H_{tj} = 1$

(Assumption 2). Thus, the forward projection converges to the measurement globally, completing the proof of Proposition 2. In RL deconvolution, each set S_k represents the entire FLFM image and is identical across iterations. Hence, the same convergence result holds for RL deconvolution.

Under Assumption 3, only the voxels within the FoV contribute to the reconstruction. Consequently, the coordinate mapping from the global coordinate system to the local FoV coordinate system ($s \rightarrow s_p$) does not affect the validity of the convergence proof.

Finally, because the KL divergence $L(x; x^*)$ is strictly convex and continuous, the only accumulation point of the sequence $\{x^k\}$ is the ground truth x^*, completing the proof.

S3 VALIDATION OF PATCH DECONVOLUTION USING SIMULATED DATA

S3.1 Simulation pipeline

To evaluate the performance of patch deconvolution under conditions where the PSF model is known, a pipeline was established to generate simulated data from high-resolution ground truth and perform reconstructions. The pipeline comprises two main components: the simulation and the reconstruction. In the simulation, high-resolution confocal ground truth data (Fig. S4a) were acquired using a confocal microscope, as described in the Methods section. Individual cells were then cropped and downsampled (Fig. S4b) to match the pixel size of the FLFM microscope at the sample plane, also detailed in the Methods section. For reconstruction comparison, the downsampled cells were convolved with the system's PSF (Eqn. S9) to generate simulated FLFM images. Each FLFM image was then reconstructed using both RL deconvolution (Fig. S4c) and patch deconvolution (Fig. S4d). The resulting RL deconvolution volumes were subsequently cropped to match the size of the corresponding patch deconvolution result.

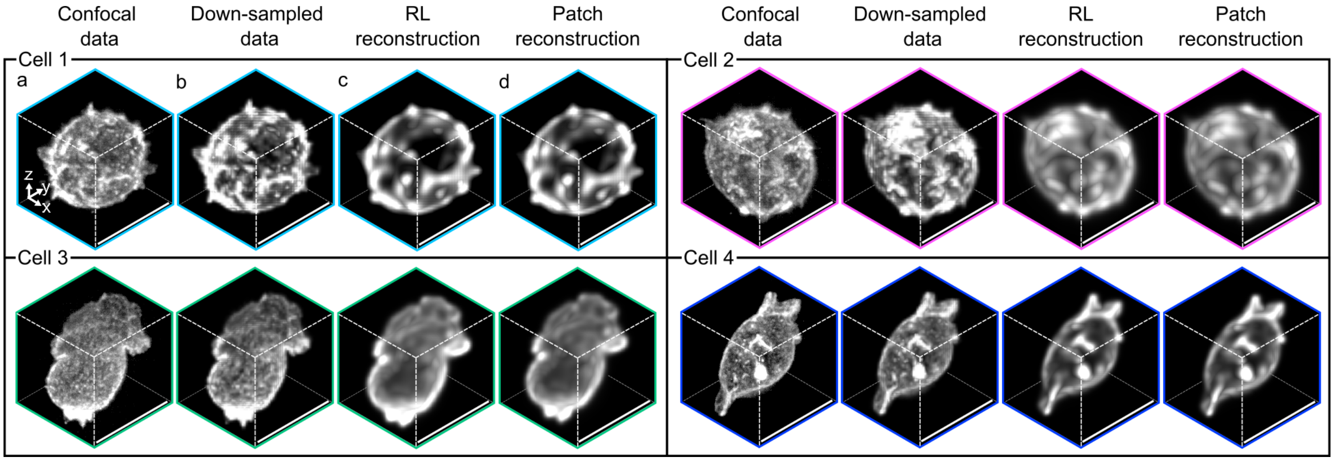

Figure S4: **Simulation pipeline gallery. a** Confocal ground truth data, **b** data down-sampled to match the pixel size of FLFM microscope, **c** RL reconstruction, and **d** patch-based reconstruction. The scale bar represents 15 µm.

S3.2 Simulated Jurkat cell reconstruction

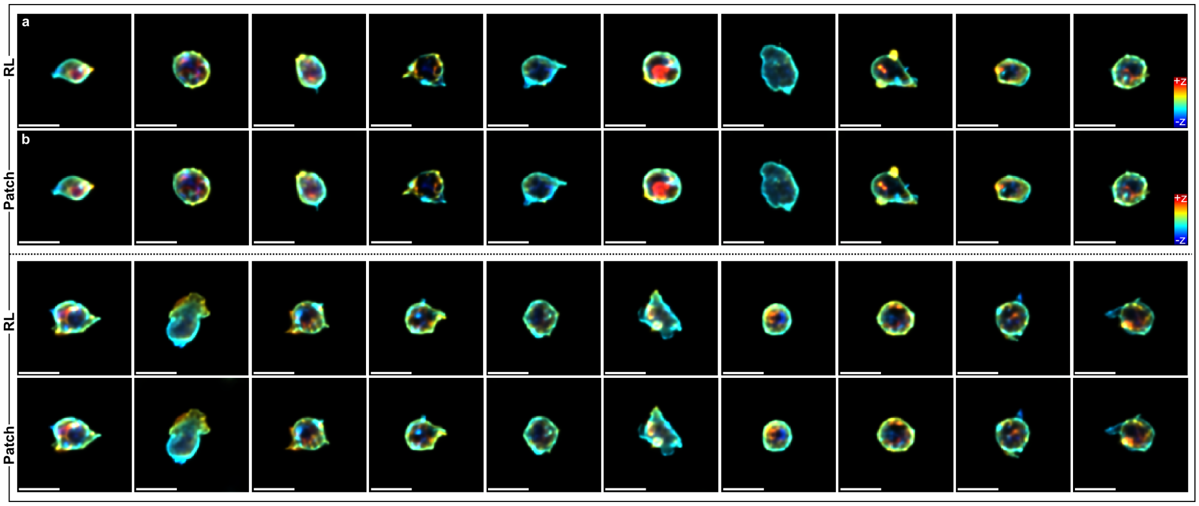

Figure S5: **Simulated Jurkat cell reconstruction gallery.** Maximum intensity projection of cells reconstructed using the RL deconvolution and patch deconvolution, showing various structural features with depth information encoded by colour. The scale bar represents 15 µm.

S3.3 Convergence of deconvolution using more iterations

As RL is a semi-convergence algorithm (6, 10), further iterations beyond the optimum lead to divergence and overfitting. To evaluate the divergence characteristics between RL and patch deconvolution, we extended the number of iterations to 250 on the same set of simulated data, rather than stopping at the number of iterations equal to the number of patches. For this analysis, the downsampled ground truth data (Fig. S4b) were used as the reference instead of selecting the 37th RL iteration as a baseline. Mean squared error (MSE) was then computed by comparing the reconstructed volumes from both RL and patch deconvolution to the downsampled ground truth. Convergence tests were performed under both high PSNR (80 dB) and typical short-exposure (11) imaging conditions (PSNR = 20 dB). At 80 dB PSNR (Fig. S6a), both RL and patch deconvolution exhibited similar convergence rates, with minimal noise and no obvious overfitting. However, at 20 dB PSNR (Fig. S6b), clear divergence occurred after approximately 50 iterations for patch deconvolution and 100 iterations for RL deconvolution. Both methods showed comparable divergence behaviour, with oscillations in the patch deconvolution MSE curve coming from the use of different patches in each iteration. Overall, the convergence and divergence of the two methods were similar.

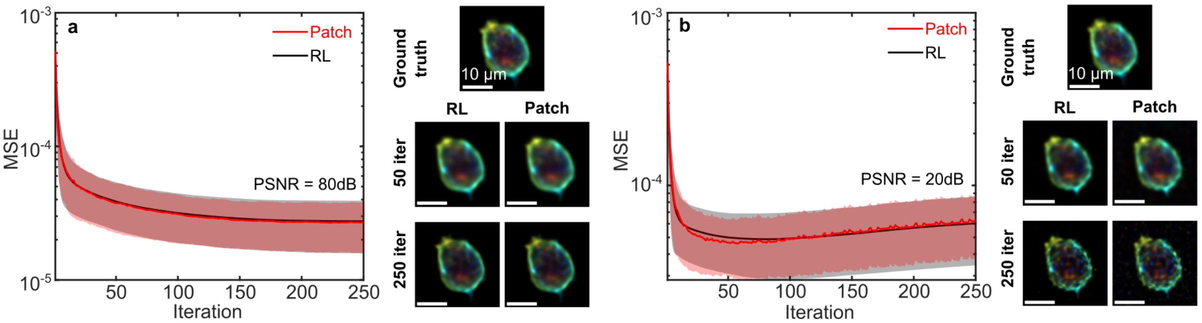

Figure S6: **Convergence comparison between RL deconvolution and patch deconvolution through larger number of iterations using MSE. a** MSE between reconstructed volumes from RL deconvolution and patch deconvolution against simulated cell data over 250 iterations with PSNR = 80 dB. **b** MSE between reconstructed volumes from RL deconvolution and patch deconvolution against simulated cell data over 250 iterations with PSNR = 20 dB, representing typical short-exposure microscopy imaging (11). Maximum intensity projections of a cell, with depth encoded by colour, are shown for both PSNR conditions and both methods. The scale bar represents 15 µm

S4 VALIDATION OF PATCH DECONVOLUTION USING EXPERIMENTAL DATA

S4.1 Bead reconstruction

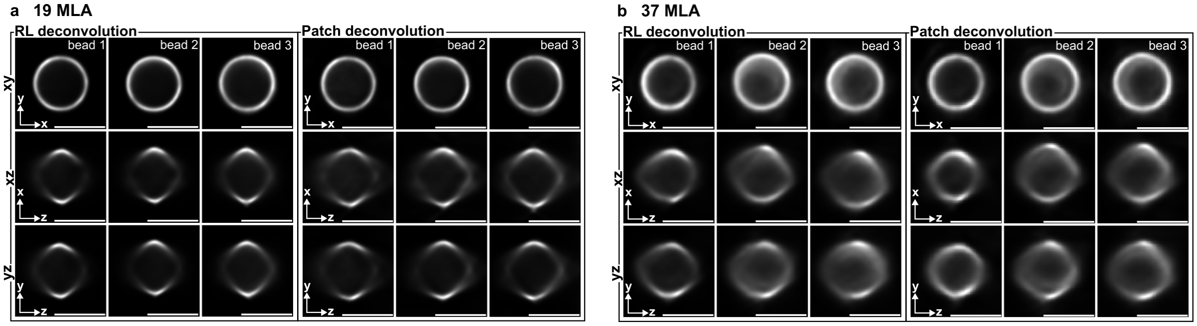

Figure S7: **Experimental bead reconstruction gallery.** Bead reconstructions from RL and patch deconvolution under 19 MLA setup **a** and 37 MLA setup **b** with a 15 µm diameter shown in the xy, xz, and yz planes. The scale bar represents 15 µm.

S4.2 Static Jurkat cell reconstruction

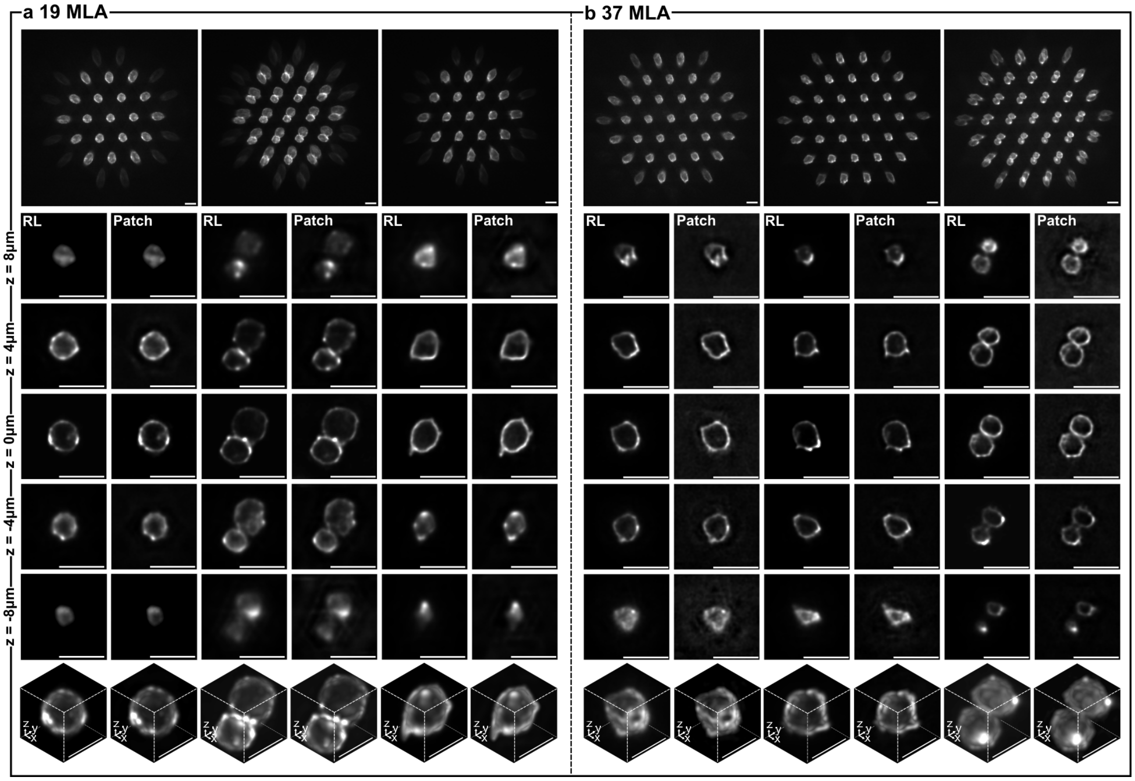

Figure S8: **Static Jurkat cell reconstruction gallery.** Reconstruction of static Jurkat cells using RL and patch deconvolution under the 19 MLA setup **a** and the 37 MLA setup **b**. Axial slices ranging from -8µm to 8 µm are shown alongside the final reconstructed 3D volumes for both methods. The scale bar represents 15 µm.

S4.3 Flow Jurkat cell reconstruction

Besides 0.5 ms exposure time in 16 bit mode discussed in the main text, we also tried 0.1 ms exposure time in 8 bit mode (Fig. S9a), equivalent to 1500 events per second, the fastest frame rate of the camera. If the FoV were cropped to 512 × 512 as stated by Hua *et al.* (5), the similar 5000 events/s acquisition speed could be achieved, which is suitable for sub-cellular imaging. 3D reconstructions remained largely consistent under 0.1 ms exposure time (Fig. S9b), though patch deconvolution exhibited higher background noise, which can be better seen in Fig. S11 from a brighter background contributed by the noise. Nevertheless, such a noise did not heavily influence the the morphology comparison and the following analysis based on the volume (Fig. S9c) even with a lower PSNR (Fig. S9d)

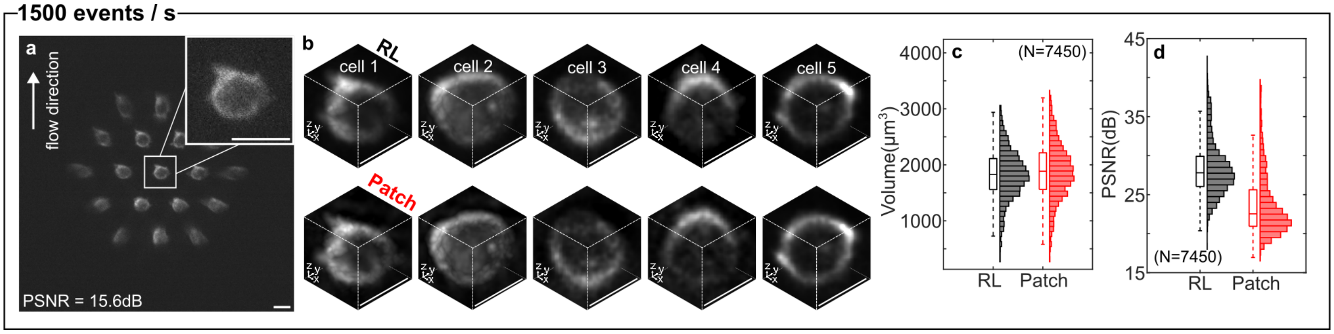

Figure S9: **Validation on flow data with 0.1 ms exposure time. a** The FLFM image with 0.1 ms exposure time from flow experiment. **b** 3D volume renderings of five cells reconstructed using patch and RL deconvolution at 0.1 ms exposure time. **c** Comparison of cell volumes from patch and RL deconvolution. **d** PSNR comparison of reconstructed volumes between patch and RL deconvolution. The scale bar represents 15 µm.

The number of events per second was determined using signals from the Kinetix camera's readout and exposure out ports (12) using a global shutter mode. A BNC cable connected these ports to an oscilloscope, where the readout port indicates digitisation status and the expose out port indicates exposure status of the camera. As shown in Fig. S10a,b, readout from the previous frame ends before the exposure of current frame finishes, and the start of the next exposure is dependent on the camera's readout time. The frame size was set to 1000 × 1000 to capture the full FLFM image for both 19 MLA and 37 MLA configurations. In 16 bit mode, the readout time was 3.54 ms, while in 8 bit mode it was reduced to 0.63 ms.

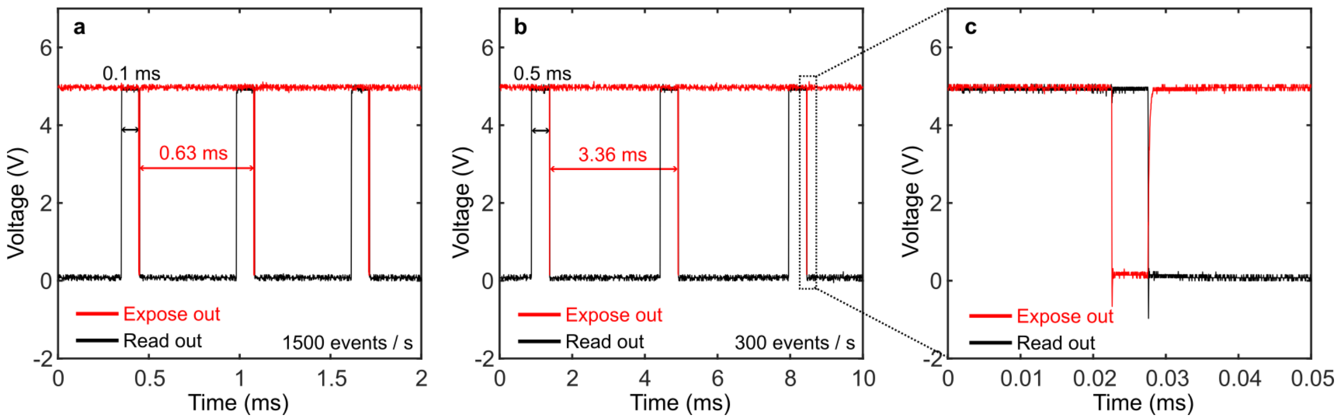

Figure S10: **Voltage output from Kinetix camera in global shutter mode. a** Voltage signals from the expose out and readout ports of the Kinetix camera at 0.1 ms exposure, operating in 8 bit mode with a frame size of 1000×1000, approximately equivalent to 1500 events per second. **b** Same signals at 0.5 ms exposure in 16-bit mode, approximately equivalent to 300 events per second. **c** Zoomed-in view showing the end of the readout and exposure periods. High voltage indicates active operation; low voltage indicates inactivity.

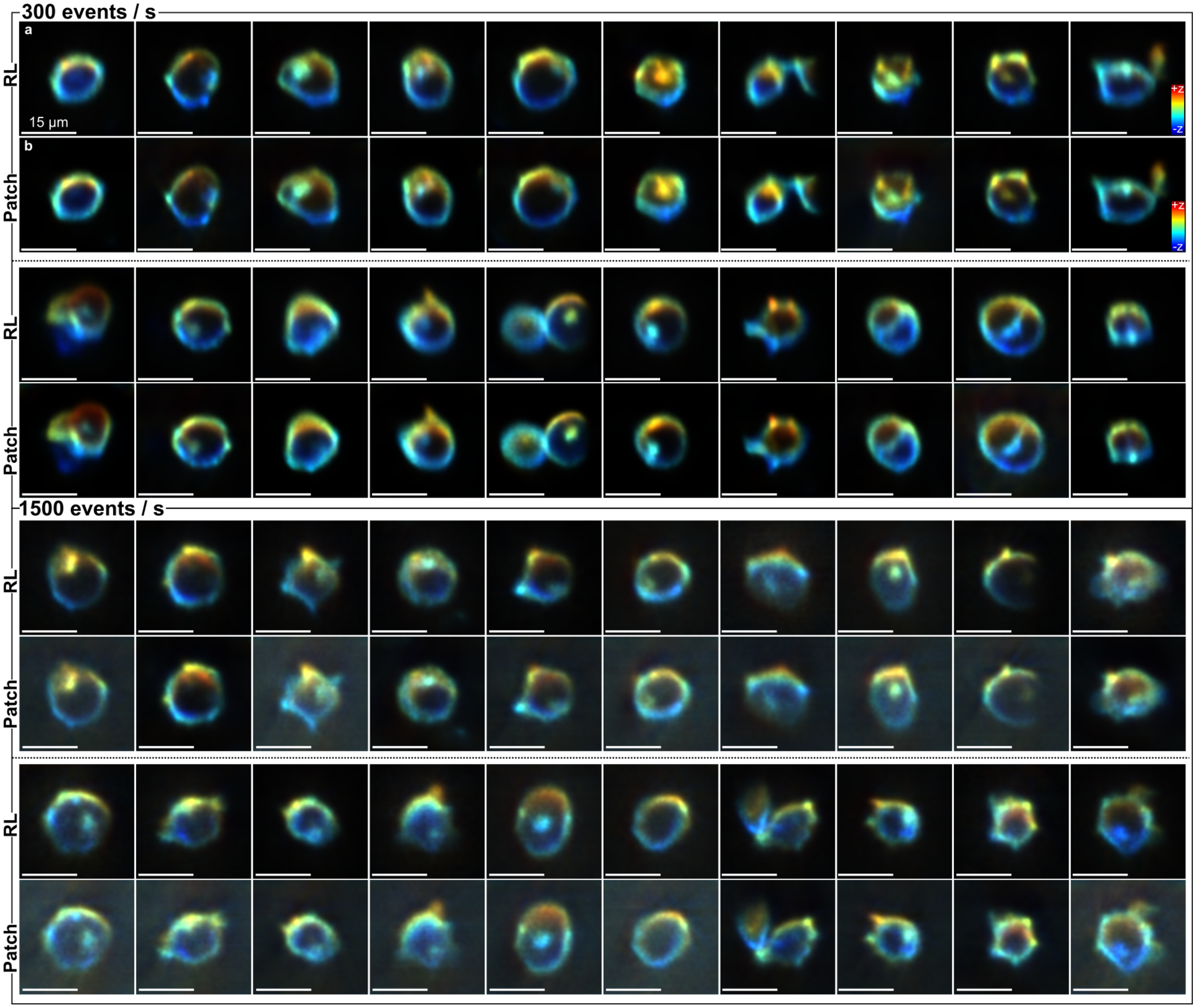

Figure S11: **Flow Jurkat cell reconstruction gallery.** Maximum intensity projection of cells reconstructed using the RL deconvolution and patch deconvolution, showing various structural features with depth information encoded by colour. The scale bar represents 15 µm.

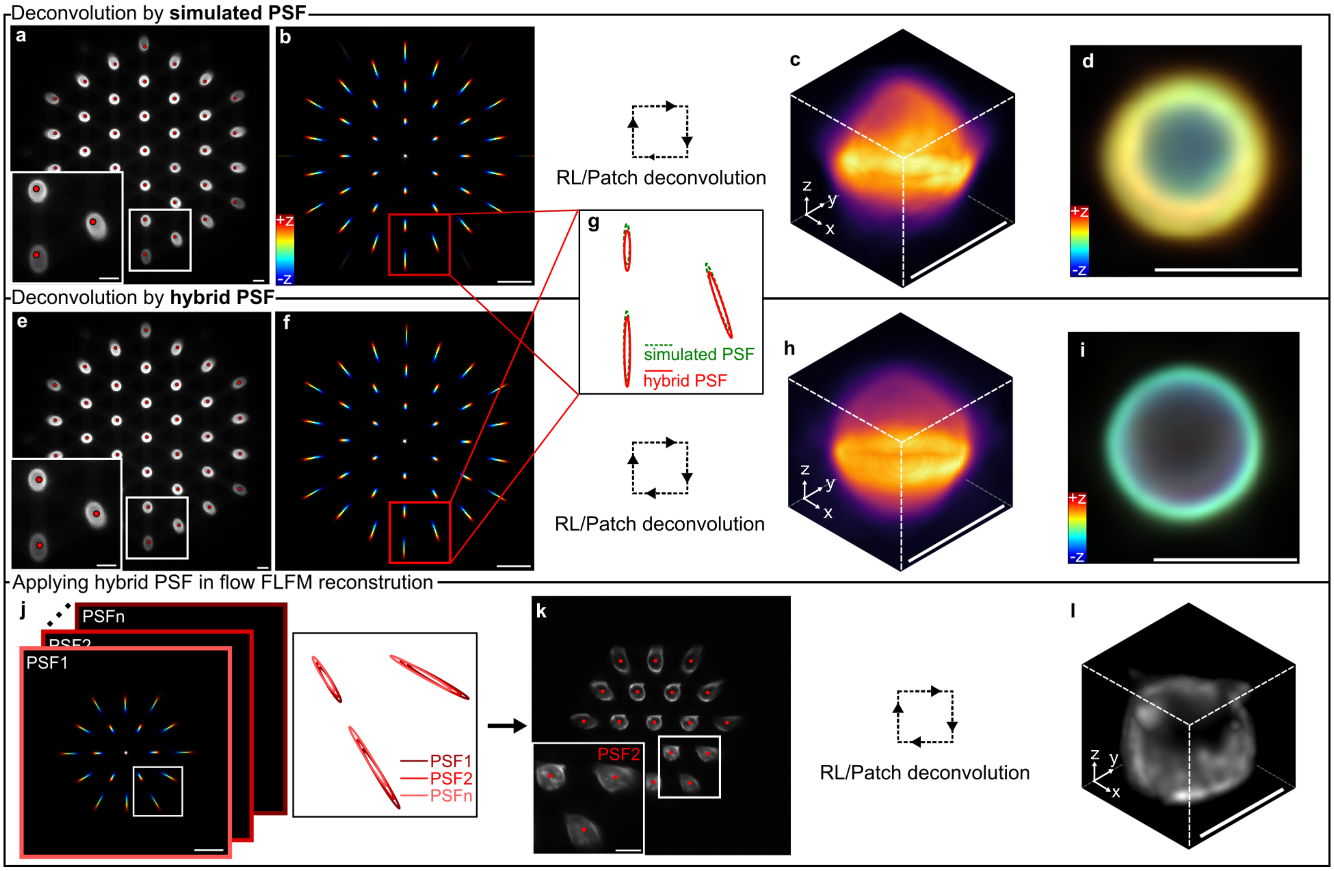

Figure S12: **Comparison between simulated PSF and hybrid PSF. a,e** FLFM image for an experimental bead image with simulated MLA centres **a** and geometric centres **e** of the bead. **b,f** Simulated PSF based on the simulated MLA centres **a** and based on geometric centres **e** (*i.e.*hybrid PSF). **c,h** 3D volumetric reconstruction of a fluorescent object by RL deconvolution using the simulated PSF **b** and hybrid PSF **f**, showing the object structure in x, y, and z dimensions. **d,i** 2D depth color-coded image of the reconstructed object **c,h**, indicating depth variations. **g** Schematic illustration comparing the 3D contour shapes of the simulated (green) and hybrid (red) PSFs, demonstrating the differences in their geometric shape. **j** The pre-simulated PSF library with different distances between views. **k** Selection of PSF from the library based on the current frame. **l** The reconstructed cell from flow using the pre-determined PSF. The scale bar represents 15 µm.

S5 IMPLEMENTATION OF PATCH DECONVOLUTION ON FLOW DATA

Both RL deconvolution and patch deconvolution algorithms, as described in Eqn. S16 and S17, rely on an accurate system PSF for reconstruction. However, various optical aberrations, including defocus from sample drifting within the microfluidic channel and spherical aberration caused by refractive index mismatches between the sample and immersion oil, can lead to discrepancies between the simulated PSF and the actual experimental FLFM data (Fig. S12a). According to Eqn. S9, misalignment between the PSF and the perspectives in the FLFM image can lead to shift of the reconstruction axially (Fig. S12d) and thus reduce the effective DoF of the reconstructed volume. This degrades the overall quality of the reconstruction (Fig. S12c,d).

To address these challenges, we adopted a hybrid PSF approach, combining experimental data into the simulated model. By adjusting the spatial positioning of the PSF based on experimental results, this method compensates for optical aberrations in the simulation model (Fig. S12g), leading to a significant improvement in the reconstruction axial position recovering (Fig. S12i) and thus the reconstruction quality (Fig. S12h). This hybrid PSF approach can produce a better reconstruction quality compared to using experimentally measured PSFs from sub-diffraction beads alone, as it offers a noise-free performance and better PSF sampling due to the higher bit depth of the simulated model.

In the flow experiment, simulating a PSF for each frame is computationally intensive. To address this, a PSF library was pre-generated by varying the pitch of the microlens, which corresponds to changes in the distance between views caused by aberrations (Fig. S12j). For each acquired frame, the average distance between views was computed, and the corresponding pre-generated PSF with the closest matching distance between views was selected for reconstruction (Fig. S12k). As a result, a satisfiable result quality (Fig. S12l) was acquired with no additional time on PSF simulation.

S6 IMPORTANCE OF 3D INFORMATION IN FLOW CYTOMETRY

We conducted a biological experiment using both conventional flow cytometry and 3D fluorescence microscopy to highlight the importance of spatial information in flow cytometry analysis. We carried out a T cell-antigen-presenting cell (APC) coculture assay using the Jurkat T cell line, which is typically evaluated using FACS, here instead focusing on spatial metrics. Jurkat cells expressing the inhibitory receptor PD-1 were cultured with APCs either with or without PD-L1 (the ligand for PD-1) to induce two different levels of activation (13). The expression of PD-1 and CD69, an activation marker, were then analysed on the Jurkat cells (Fig. S13a). While these proteins are known to be expressed at the plasma membrane, spatial information remains unexplored, particularly at the population scale.

Activated Jurkat cells were first analysed using a conventional flow cytometer. After standard gating, the intensity distributions from forward scatter (FSC) and red laser channel (RL1) were obtained (Fig. S13b), revealing two clusters in RL1 consistent with the expected medium and high CD69 expression. The same samples were then imaged using a confocal microscope. Data were down-sampled, transformed into FLFM images, and reconstructed using patch deconvolution (Fig. S4). The mean fluorescence intensity (MFI) was computed by averaging voxel values within the binary mask of each cell, and cell volume was determined based on the number of voxels within the filled cell body mask. Fig. S13c shows a scatter plot of MFI versus volume, revealing two distinct clusters, consistent with results obtained from conventional flow cytometry.

To further leverage 3D spatial information, we calculated the spatial correlation between CD69 and PD-1 distributions (Fig. S14d) on cells with negative PD-L1 binding (*i.e.*high intensity in both PD-1 and CD69 channel), where values approaching 1 indicate strong colocalisation, and values near 0 indicate no spatial association. Most Jurkat cells exhibited strong spatial correlation (>0.9) between CD69 and PD-1 (Fig. S14e). However, cells 1 (blue) and 2 (pink), which had similar volume and intensity values (Fig. S13f) and were therefore indistinguishable in the MFI vs. volume plot (Fig. S13g), showed markedly different spatial correlations (cell 1 = 0.23; cell 2 = 0.97). A gallery of cells with similar MFI values but differing spatial correlation is shown in Fig. S14. This example illustrates the power of 3D spatial information in identifying cellular features that conventional flow cytometry methods overlook. Such capabilities could enable the detection of rare spatial events or distinct subpopulations, providing new dimensions for analysing heterogeneous cellular responses and complex cell behaviours.

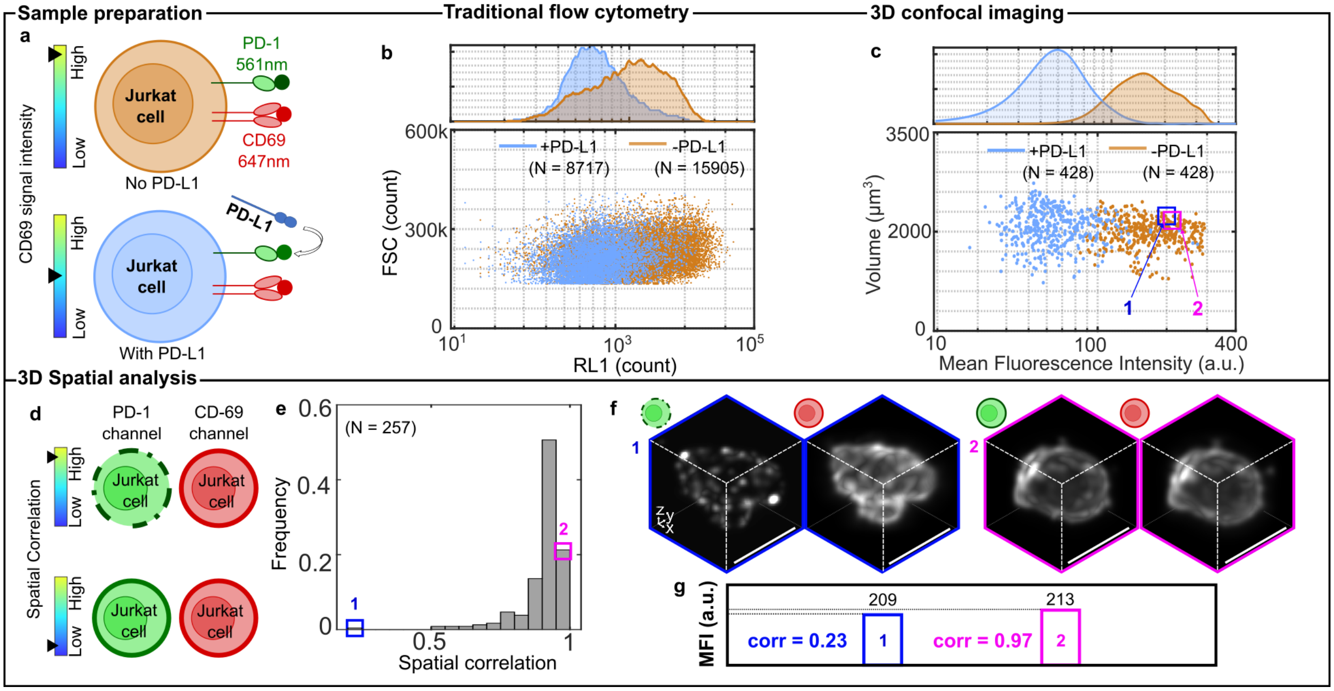

Figure S13: **Importance of 3D information in flow cytometry. a** Jurkat cells transduced with PD-1 with and without PD-L1 binding. CD69 expression indicates cell activation level. **b** FSC vs RL1 plot compares PD-L1 positive (orange) and negative (blue) cells after gating. **c** Comparison of cell volume and mean fluorescence intensity (MFI) for +PD-L1 (orange) and -PD-L1 (blue) cells using a confocal microscope. **d** 3D spatial correlation analysis where low spatial correlation indicates different membrane distributions of PD-1 and CD69 on the cell surface. **e** Histogram showing spatial correlation results for Jurkat cells without PD-L1 binding and with signals in both CD69 and PD-1 channels. **f** Cell (1) shows low spatial correlation (corr = 0.23) while cell (2) shows high spatial correlation (corr = 0.97). **g** Box plots showing MFI values for PD-1 and CD69 for the two example cells. The scale bar represents 15 µm.

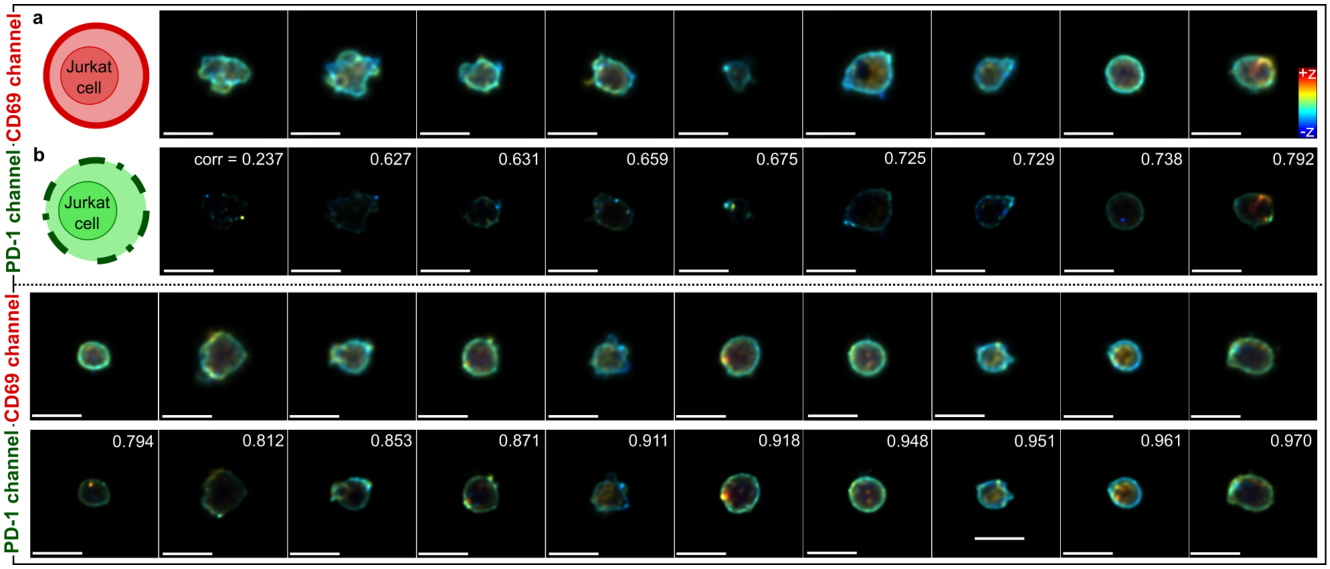

Figure S14: **CD69 and PD-1 spatial correlation gallery.** Maximum intensity projection of reconstructed volume in CD69 channel (**a**, top row) and PD-1 channel (**b**, bottom row) with depth information encoded by colour. The scale bar represents 15 µm.

REFERENCES

1. Guo, C., W. Liu, X. Hua, H. Li, and S. Jia, 2019. Fourier light-field microscopy. *Optics Express* 27:25573.

2. Galdón, L., G. Saavedra, J. Garcia-Sucerquia, M. Martínez-Corral, and E. Sánchez-Ortiga, 2022. Fourier lightfield microscopy: a practical design guide. *Applied Optics* 61:2558.

3. Sims, R. R., S. A. Rehman, M. O. Lenz, S. I. Benaissa, E. Bruggeman, A. Clark, E. W. Sanders, A. Ponjavic, L. Muresan, S. F. Lee, and K. O'Holleran, 2020. Single molecule light field microscopy. *Optica* 7:1065.

4. Born, M., E. Wolf, A. B. Bhatia, P. C. Clemmow, D. Gabor, A. R. Stokes, A. M. Taylor, P. A. Wayman, and W. L. Wilcock, 1999. Principles of Optics: Electromagnetic Theory of Propagation, Interference and Diffraction of Light. Cambridge University Press, 7 edition.

5. Hua, X., K. Han, B. Mandracchia, A. Radmand, W. Liu, H. Kim, Z. Yuan, S. M. Ehrlich, K. Li, C. Zheng, J. Son, A. D. S. Trenkle, G. A. Kwong, C. Zhu, J. E. Dahlman, and S. Jia, 2024. Light-field flow cytometry for high-resolution, volumetric and multiparametric 3D single-cell analysis. *Nature Communications* 15.

6. Dey, N., L. Blanc-Feraud, C. Zimmer, P. Roux, Z. Kam, J. C. Olivo-Marin, and J. Zerubia, 2006. Richardson-Lucy algorithm with total variation regularization for 3D confocal microscope deconvolution. *Microscopy Research and Technique* 69:260–266.

7. Richardson, W. H., 1972. Bayesian-Based Iterative Method of Image Restoration. *JOURNAL OF THE OPTICAL SOCIETY OF AMERICA* 62:55–59.

8. Lucy, L. B., 1974. An iterative technique for the rectification of observed distributions. *THE ASTRONOMICAL JOURNAL* 79:745–754.

9. Biggs, D. S. C., and M. Andrews, 1997. Acceleration of iterative image restoration algorithms. *Applied Optics* 36:1766–1775.

10. Hudson, H. M., and R. S. Larkin, 1994. Accelerated Image Reconstruction Using Ordered Subsets of Projection Data. *IEEE TRANSACTIONS ON MEDICAL IMAGING* 13:601–609.

11. Chen, F., J. Liu, D. Gou, X. Zhang, L. Chen, and H. Liao, 2020. An accurate and universal approach for short-exposure-time microscopy image enhancement. *Computerized Medical Imaging and Graphics* 83.

12. Teledyne Photometrics, 2021. Kinetix Camera Manual. Teledyne Vision Solutions / Teledyne Photometrics. https://www.photometrics.com/wp-content/uploads/2021/11/Kinetix-Manual-58-723-004_RevA01.pdf, rev. A01; PDF available online.

13. Patsoukis, N., Q. Wang, L. Strauss, and V. A. Boussiotis, 2020. Revisiting the PD-1 pathway. *Sci. Adv* 6.
